# Functional architecture of neural circuits for leg proprioception in *Drosophila*

**DOI:** 10.1016/j.cub.2021.09.035

**Published:** 2021-10-11

**Authors:** Chenghao Chen, Sweta Agrawal, Brandon Mark, Akira Mamiya, Anne Sustar, Jasper S. Phelps, Wei-Chung Allen Lee, Barry J. Dickson, Gwyneth M. Card, John C. Tuthill

**Affiliations:** 1 Department of Physiology and Biophysics, University of Washington, 1705 N.E. Pacific Street, Seattle, WA 98195, USA; 2 Janelia Research Campus, Howard Hughes Medical Institute, 19700 Helix Drive, Ashburn, VA 20147, USA; 3 Department of Neurobiology, Harvard Medical School, 220 Longwood Avenue, Boston, MA 02115, USA; 4 Present address: Queensland Brain Institute, University of Queensland, St. Lucia, QLD 4072, Australia; 5 These authors contributed equally; 6 Twitter: @casa_tuthill; 7 Lead contact

## Abstract

To effectively control their bodies, animals rely on feedback from proprioceptive mechanosensory neurons. In the *Drosophila* leg, different proprioceptor subtypes monitor joint position, movement direction, and vibration. Here, we investigate how these diverse sensory signals are integrated by central proprioceptive circuits. We find that signals for leg joint position and directional movement converge in second-order neurons, revealing pathways for local feedback control of leg posture. Distinct populations of second-order neurons integrate tibia vibration signals across pairs of legs, suggesting a role in detecting external substrate vibration. In each pathway, the flow of sensory information is dynamically gated and sculpted by inhibition. Overall, our results reveal parallel pathways for processing of internal and external mechanosensory signals, which we propose mediate feedback control of leg movement and vibration sensing, respectively. The existence of a functional connectivity map also provides a resource for interpreting connectomic reconstruction of neural circuits for leg proprioception.

## In brief

To understand how diverse proprioceptive signals from the *Drosophila* leg are integrated by downstream circuits, Chen et al. use optogenetics and calcium imaging to map functional connectivity between sensory and central neurons. This work identifies parallel neural pathways for processing leg vibration vs. joint position and movement.

## Graphical abstract

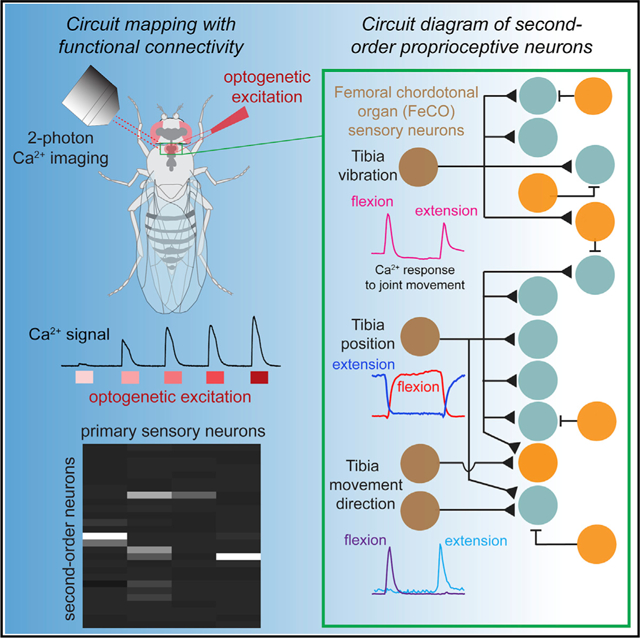

## INTRODUCTION

Proprioception, the sense of limb position and movement, plays an indispensable role in motor control by providing continuous sensory feedback to motor circuits in the central nervous system. Proprioception is important for inter-leg coordination during locomotion,^1,2^ stabilization of body posture,^3,4^ and motor learning.^5,6^ Loss of limb proprioception impairs locomotion and motor control.^7,8^ Thus, mapping neural circuits that process proprioceptive information is a prerequisite to understanding the role of proprioception in motor flexibility and recovery from injury.

Proprioception relies on mechanosensory neurons embedded in joints and muscles throughout the body, which are referred to as proprioceptors. Different types of proprioceptors detect distinct features of body kinematics. In vertebrates, Golgi tendon organs detect mechanical load on the body, and muscle spindles encode muscle fiber length and contraction velocity.^9,10^ Proprioceptors in invertebrates detect similar features. The three predominant classes of proprioceptors in insects are campaniform sensilla, hair plates, and chordotonal neurons.^11^ Dome-shaped campaniform sensilla encode mechanical load by detecting strain in the cuticle,^4^ hair plates act as joint limit detectors,^12^ and chordotonal neurons detect multiple features of joint kinematics.^13,14^ Although they differ in structure, the common functional properties of vertebrate and invertebrate proprioceptors suggest that they have convergently evolved to encode similar mechanical features.^9^

Compared to other primary senses, the organization of central circuits for leg proprioception remains poorly understood. Pioneering work in larger insect species, such as the locust^2^ and stick insect,^15^ characterized the anatomy and physiology of central proprioceptive neurons. However, most of this prior work relied on sharp-electrode recordings from single neurons, which made it challenging to understand how they operate collectively as a circuit to control behavior. Understanding circuit-level architecture and function is aided by the existence of genetic tools to label, manipulate, and record from identified classes of neurons. Such genetic tools have recently become available for proprioceptive circuits in the *Drosophila* ventral nerve cord (VNC), the invertebrate analog of the spinal cord.^16^ An additional advantage of *Drosophila* is the existence of an electron microscopy (EM) volume of the adult VNC,^17^ which enables synapse-level reconstruction of VNC circuits. Together, the combination of genetic tools and connectomics data provides an opportunity to link connectivity and function of central circuits for leg proprioception.

**Figure 1.**
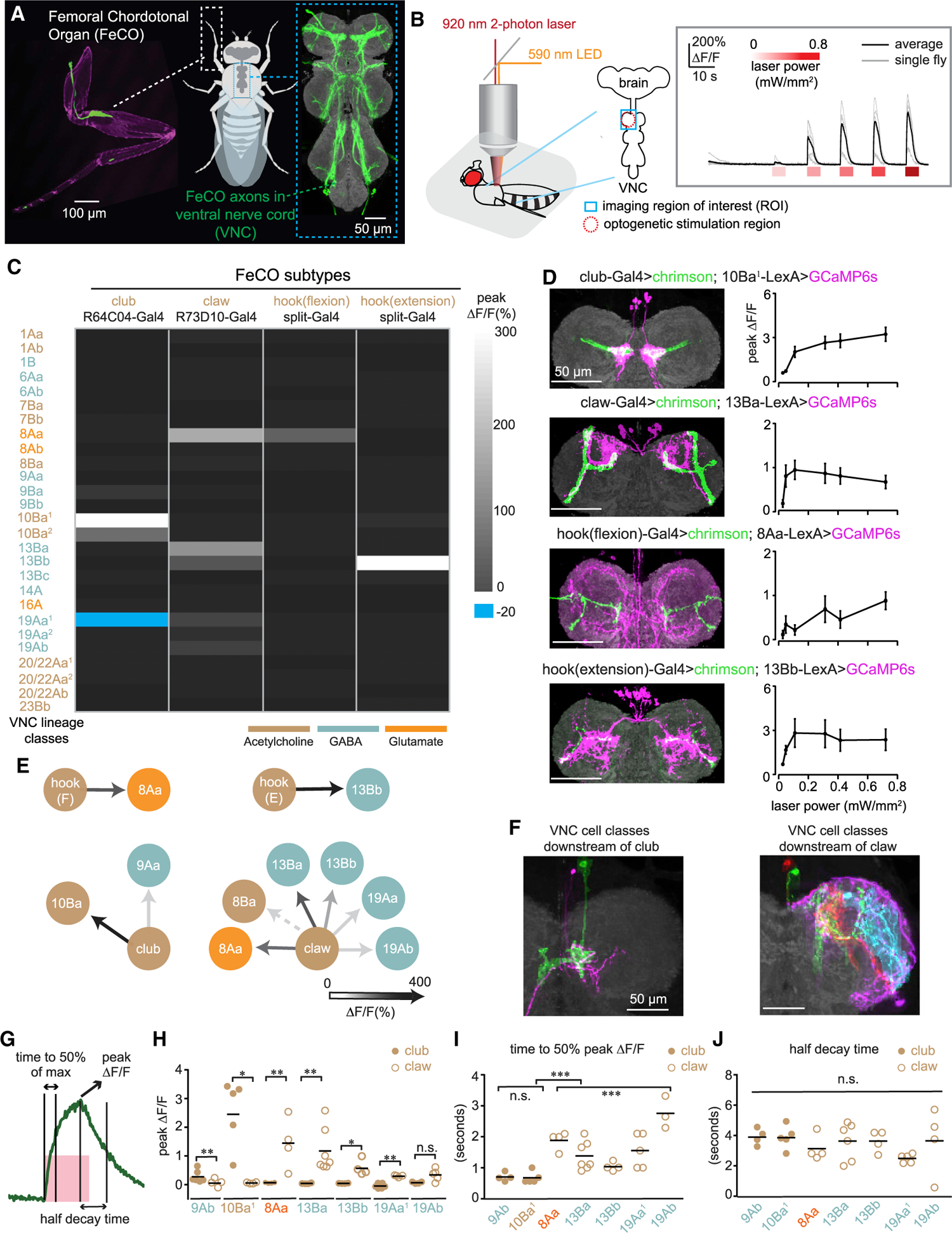
Building a functional connectivity map between FeCO sensory neurons and central neurons in the fly VNC (A) (Left) A confocal image of the foreleg (T1) of *Drosophila melanogaster.* The FeCO cell bodies (left) and axons (right) are labeled by GFP (green) driven by *iav*-Gal4. Cuticle auto-fluorescence is magenta (left), and the VNC neuropil stained by nc82 is shown in gray (right). (B) Experimental setup for two-photon calcium imaging from VNC neurons while optogenetically stimulating FeCO axons. (Left) Schematic of experimental setup is shown. The blue window indicates the imaging region (region of interest [ROI]) and red dashed circle indicates the region of optogenetic stimulation. (Right) Example traces of GCaMP6s fluorescence in 10Ba^1^ neurons in response to optogenetic activation of club neurons (n = 6 flies) are shown. The red bars below the traces indicate the 5-s stimulation window and intensity. (C) A heatmap summarizing the average peak calcium signal (ΔF/F) in VNC neurons following optogenetic activation of each FeCO subtype (n ≥ 4 flies). The colors for each lineage and FeCO subtype indicate the putative neurotransmitter that they release. Superscript numbers indicate independent LexA lines that label the same lineage; genotypes are listed in Figure S2. (D) Anatomy (left) and peak calcium responses (right; mean ± SEM) of each sensory and central neuron pair (n = 6, 7, 4, and 5 flies). (E) A summary of the predominant targets downstream of each FeCO subtype. Functional connectivity strength is indicated by the shading of the arrow. Note that the functional connectivity between claw and 8Ba neurons varied across flies (Figures S3 and S4), while other responses were consistent. (F) Single neuron anatomy from each neuron class downstream of club (left) and claw (right) sensory neurons was aligned to a common VNC template. (G) Quantification of calcium response kinetics. The pink window indicates 5-s stimulus duration. The green curve is an example calcium trace. (H–J) Peak calcium response (H; ΔF/F; *p < 0.05; **p < 0.01; n.s., no significant difference; Mann-Whitney test), time to 50% of the maximum signal (I; *p < 0.05; **p < 0.01; Mann-Whitney test and Kruskal-Wallis test), and time to 50% decay from the max for neurons downstream of club (solid brown dots) and claw (open brown circles) sensory neurons (J; Kruskal-Wallis test). Each point represents data from an individual fly. Bars indicate the average across flies.

The largest proprioceptive organ in the *Drosophila* leg is the femoral chordotonal organ (FeCO), which is composed of ~152 mechanosensory neurons^18^ located in the proximal femur and attached to the tibia by a series of tendons (Figure 1A). Calcium imaging has revealed that *Drosophila* FeCO neurons can be divided into three basic subtypes: claw neurons encode tibia position; hook neurons encode movement direction; and club neurons encode bidirectional movement and vibration.^19^ The axons of each subtype project to distinct regions of the VNC. This organization suggests that signals from different FeCO subtypes may be processed by separate downstream neurons (Figure 1A, right). However, apart from three specific VNC cell classes,^20^ little is known about how information from different FeCO subtypes is integrated by downstream circuits in the *Drosophila* VNC that underlie sensation and guide movement of the leg.

In this study, we investigate the logic of sensory integration within leg proprioceptive circuits of the *Drosophila* VNC. We first combined two-photon calcium imaging of second-order VNC neurons with optogenetic stimulation of specific FeCO subtypes. This strategy, named “functional connectivity,” has previously been used to map the structure of visual^21^ and navigation^22^ circuits in *Drosophila*. Our functional connectivity analysis identified separate circuits for processing tibia vibration vs. tibia position and movement. We further analyzed spatial and multimodal integration in three specific classes of central neurons and validated their response properties during leg movement. Using spatially targeted optogenetic stimulation to map receptive-field structure, we found that each class either integrates sensory information from multiple FeCO subtypes or from the same FeCO subtype across multiple legs. Finally, we find that inhibition sculpts the adaptation dynamics of second-order neurons encoding leg movement and vibration. Our results demonstrate that diverse proprioceptive signals from different sensory neuron subtypes and locations on the body are directly integrated by second-order neurons and reveal separate central pathways for processing of external substrate vibration and internal, self-generated leg joint kinematics.

## RESULTS

### Functional connectivity identifies second-order proprioceptive neurons in the fly VNC

We began by creating genetic driver lines to specifically manipulate the activity of each FeCO subtype with optogenetics. Using an anatomical screen of existing driver lines,^23,24^ we created intersectional Split-Gal4 lines that specifically label club, claw, and hook neurons (Figure S1A), which we previously found encode tibia movement and vibration, position, and direction.^19^ To measure the proprioceptive tuning of the neurons labeled by each Split-Gal4 line, we used two-photon calcium imaging while swinging the tibia between flexion and extension (Figures S1D and S1E). In addition to confirming the proprioceptive encoding of each subtype, these experiments identified a new FeCO subtype that responds to tibia extension in a directionally tuned manner. The projections of these neurons are slightly different from the flexion-tuned hook neurons (Figure S1E); therefore, we refer to this new FeCO subtype as “hook (extension)” and flexion-tuned hook neurons as “hook (flexion).”

With improved tools to specifically target FeCO subtypes, we next sought to identify their downstream partners in the VNC. The VNC is composed of about 20,000 neurons that develop from 34 hemilineages.^25^ Neurons within a hemilineage are anatomically^26^ and transcriptionally^27,28^ similar; they also use the same primary neurotransmitter.^29^ By visually screening a collection of LexA driver lines, we identified 27 LexA lines that sparsely labeled VNC neurons from each hemilineage that anatomically overlapped with FeCO axons (Figure S2). We denote driver lines that label different neuron classes within the same hemilineage using lowercase letters, e.g., 9Aa, 9Ba, etc.

In *Drosophila*, most neurons release one of three canonical neurotransmitters: acetylcholine; GABA; and glutamate.^27,30^ Fast excitation is primarily mediated by acetylcholine; inhibition is mediated by GABA and glutamate. We were able to infer the neurotransmitter released by each VNC neuron class (Figures 1C, 1E, and S1), based on their hemilineage identity.^29^

We imaged calcium signals from each LexA line in the VNC with GCaMP6s^31^ while optogenetically stimulating the axons of FeCO neurons expressing the ChR2 variant Chrimson (Figure 1B).^32^ To account for differences in response threshold and synaptic strength, we tested a range of stimulation intensities (Figure 1B). Calcium signals evoked by optogenetic stimulation typically plateaued at a stimulus intensity of 0.3 mW/mm^2^ (Figures 1B–1D). Importantly, calcium responses of FeCO axons to optogenetic stimulation at this intensity were of similar amplitude to calcium responses during passive leg movements (Figures S1C and S1E). Therefore, we used this stimulus intensity for subsequent group analyses.

Overall, we identified 8 classes of VNC neurons from 6 lineages that responded to optogenetic stimulation of one or more subtypes of FeCO neurons: 8Aa; 8Ba; 9Ba; 10Ba; 13Ba; 13Bb; 19Aa; and 19Ab (Figure 1C). Of these, 2 neuron classes (9Ba and 10Ba) responded to stimulation of club neurons. The remaining 6 classes responded consistently to the stimulation of claw neurons (Figure 1C). In addition to responding to claw neurons, 8Aa and 13Bb neurons responded to stimulation of hook (flexion) and hook (extension) neurons, respectively.

To estimate the completeness of our functional connectivity screen, we compared our results to trans-synaptic labeling with *trans*-Tango.^33^ We used Split-Gal4 driver lines to express the *trans*-Tango ligand in each FeCO subtype, and the *trans-*Tango receptor was expressed in all neurons (Figure S1B). In total, we counted 1,053 putative second-order neurons in the VNC labeled by *trans*-Tango (club: 216, claw: 566, hook (flexion): 74, and hook (extension): 197), compared to 700 cells labeled by functional connectivity (club: 147, claw: 443, hook (flexion): 21, and hook (extension): 89; see Table S1 for more details). Overall, our functional connectivity screen identified ~1/3 fewer postsynaptic cells than *trans*-Tango. However, due to the fact that the driver lines used did not label all FeCO neurons and the limited sensitivity of *trans*-Tango, we expect these analyses underestimate the total number of VNC cells postsynaptic to FeCO axons.

The amplitude of calcium signals driven by optogenetic stimulation of a particular FeCO subtype (e.g., club or claw) varied across different downstream neurons (Figure 1H). Neuron classes that responded to stimulation of club and claw axons were non-overlapping: neurons downstream of the club occupy a medial region of the VNC (mVAC), whereas neurons downstream of the claw arborize more laterally or in the intermediate neuropil (IntNp) (Figure 1F). Comparing calcium dynamics of VNC neurons during proprioceptor excitation revealed that VNC neurons downstream of the club had a faster time to peak than those downstream of the claw (Figure 1I). In contrast, the decay of the calcium response of these two groups (club and claw targets) was similar (Figure 1J). These differences may reflect distinct temporal dynamics in pathways that process leg position versus vibration-related signals.

In summary, our results reveal the first steps of proprioceptive processing downstream of the FeCO. Signals from club (vibration) and claw (position) are routed to different downstream targets, while claw (position) and hook (direction and movement) signals are combined. We also observed interesting differences in the response dynamics of neurons downstream of the club and claw, consistent with their roles in encoding high and low frequencies, respectively.

### 9Ba, a VNC cell class downstream of club axons, integrates bidirectional movements and vibration signals from contralateral legs

The two cell classes we identified as downstream targets of the club both have contralateral projections that cross the VNC midline. We therefore hypothesized that they integrate club signals across multiple legs.

**Figure 2.**
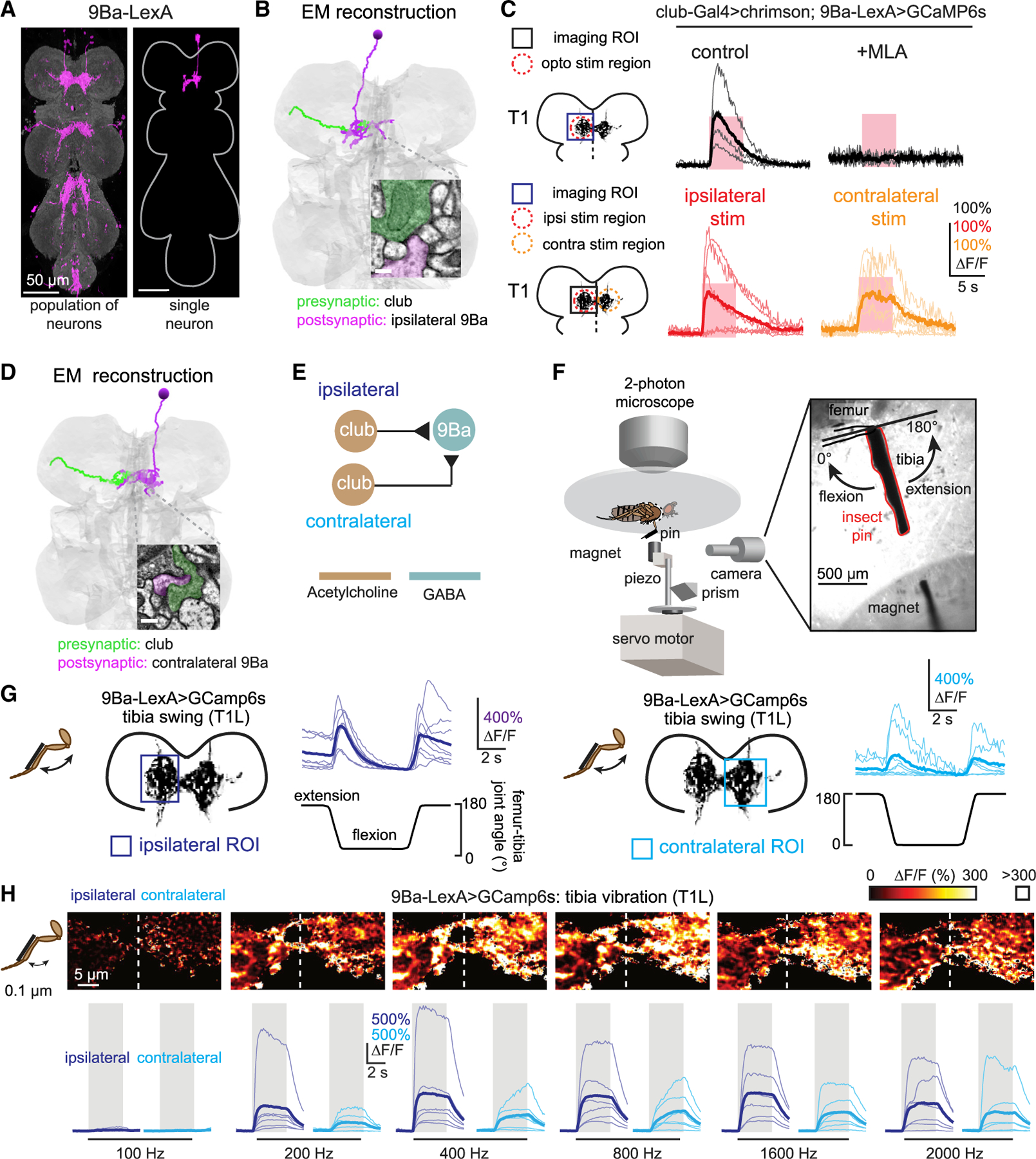
9Ba neurons receive bidirectional movement and vibration signals from club neurons across both front legs (A) Anatomy of 9Ba neurons. Magenta is GFP driven by *R18H03-LexA*; neuropil was stained with nc82 (gray). A single 9Ba neuron (magenta) is labeled by multi-color FLPout. Both images were aligned to a common VNC template. (B) Anatomical reconstruction from EM showing an example of a 9Ba neuron (magenta) that receives direct synaptic input from an ipsilateral club axon (green). The inset shows an example of a synapse between the two cells. Scale bar represents 200 nm. (C) Calcium response of 9Ba neurons to optogenetic stimulation of club neurons. Top: calcium responses of 9Ba neurons in the left prothoracic VNC (T1L) to stimulation of the axons from club neurons in the left foreleg (T1L; n = 4) are shown. Methyllycaconitine (MLA) (1 μM; n = 4) effectively blocks excitation from club neurons. Bottom: calcium responses of 9Ba in left neuromeres of the prothoracic VNC to optogenetic stimulation of club axons in T1L (indicated by the red dashed circle; n = 5) and T1R (orange; n = 6) are shown. The pink regions indicate stimulus duration (5 s; laser power = 0.28 mW/mm^2^). (D) Same as in (B) but showing direct connection between a contralateral 9Ba neuron (magenta) and a club axon (green) traced from the EM volume. Scale bar represents 200 nm. (E) Proposed wiring diagram for how club axons connect to 9Ba neurons. (F) Experimental setup for calcium imaging during passive leg movements. Two-photon calcium imaging was used to record calcium signals from the central VNC neurons while controlling and tracking the femur-tibia joint. A pin was glued to the tibia of the front leg and manipulated using a magnet mounted on a motor. The joint was tracked with high-speed video. (G) 9Ba neurons respond to ipsilateral (n = 6) and contralateral (n = 6) passive tibia movement. Thin lines are recordings from individual flies; the thicker line indicates the average across flies. (H) 9Ba neurons respond to 0.1 μm vibration of both the ipsi- (n = 6) and contralateral (n = 6) tibia. Top: the majority of pixels had a ΔF/F value between 0% and 300%; outlier pixels with a value above 300% ΔF/F were set to white for visualization purposes. Bottom: calcium signals in 9Ba neurons during tibia vibration across different frequencies are shown. Thin lines are calcium signals from individual flies; thicker line indicates the average across flies.

We first examined connectivity between club axons and 9Ba neurons, a class of GABAergic interneurons local to each VNC segment (Figure 2A, left). Single 9Ba neurons densely innervate the ipsilateral neuromere, but also extend a process contralaterally, across the midline (Figure 2A, right). Identification and reconstruction of 9Ba and club neurons in an EM volume of the *Drosophila* VNC^17^ revealed the existence of direct synaptic connections between club axons and ipsilateral 9Ba neurons (Figure 2B; Table S2). Signal transmission between FeCO and central neurons may be mediated by chemical synapses, electrical gap junctions, or a mixture of the two. Because FeCO neurons release acetylcholine,^19^ we used an antagonist of nicotinic acetylcholine receptors (methyllycaconitine [MLA], 1 μM) to test for the presence of electrical signaling mediated by gap junctions. MLA blocked club-driven calcium signals in 9Ba (Figure 2C, top), suggesting that the connection between club and 9Ba neurons is mediated by acetylcholine receptors.

To ask whether 9Ba neurons integrate club signals from multiple legs, we measured calcium responses from 9Ba neurons in the left prothoracic (T1) neuromere while stimulating club axons from the left (ipsilateral) or right (contralateral) T1 legs using spatially targeted optogenetic excitation. 9Ba neurons in the left T1 neuromere increased their calcium activity in response to excitation of club axons from either left or right T1 legs (Figure 2C, bottom). These data suggest that 9Ba neurons integrate ipsilateral and contralateral signals from club neurons (Figure 2E). EM reconstruction of a 9Ba neuron with a cell body in the right T1 neuromere confirmed the existence of direct synaptic input from contralateral club axons (Figure 2D; Table S2).

To understand how 9Ba neurons encode leg movements *in vivo*, we recorded 9Ba calcium signals while manipulating the femur-tibia joint of the fly’s left leg with a magnetic control system (Figure 2F).^19^ We observed phasic calcium signals of 9Ba neurons in both ipsilateral and contralateral neuromeres, in response to passive tibia flexion and extension (Figure 2G). Similar to what we observed in the club Split-Gal4 line (Figure S1D), 9Ba also exhibited higher baseline activity when the tibia was held at full extension compared to flexion; inspection of high-speed video suggests that this response is caused by vibrations produced by the fly’s resistance to passive tibia extension. Consistent with this hypothesis, 9Ba neurons responded strongly to low-amplitude (0.1 μm) vibration of the tibia (Figure 2H) at frequencies from 200 to 2,000 Hz, similar to the club neuron population.^19^ Thus, 9Ba neurons encode high-frequency, low-amplitude movement of the tibia, consistent with a role for sensing external substrate vibrations.

In summary, GABAergic 9Ba neurons integrate club signals from left and right legs in the same segment to encode tibia movement and high-frequency vibration (Figure 2E). 9Ba neurons are thus positioned to provide inhibition to other neurons in the vibration-processing pathway or to mediate interactions between movement and vibration pathways.

### 10Ba, a VNC cell class downstream of the club, integrates bidirectional movements and vibration signals from multiple legs across segments

**Figure 3.**
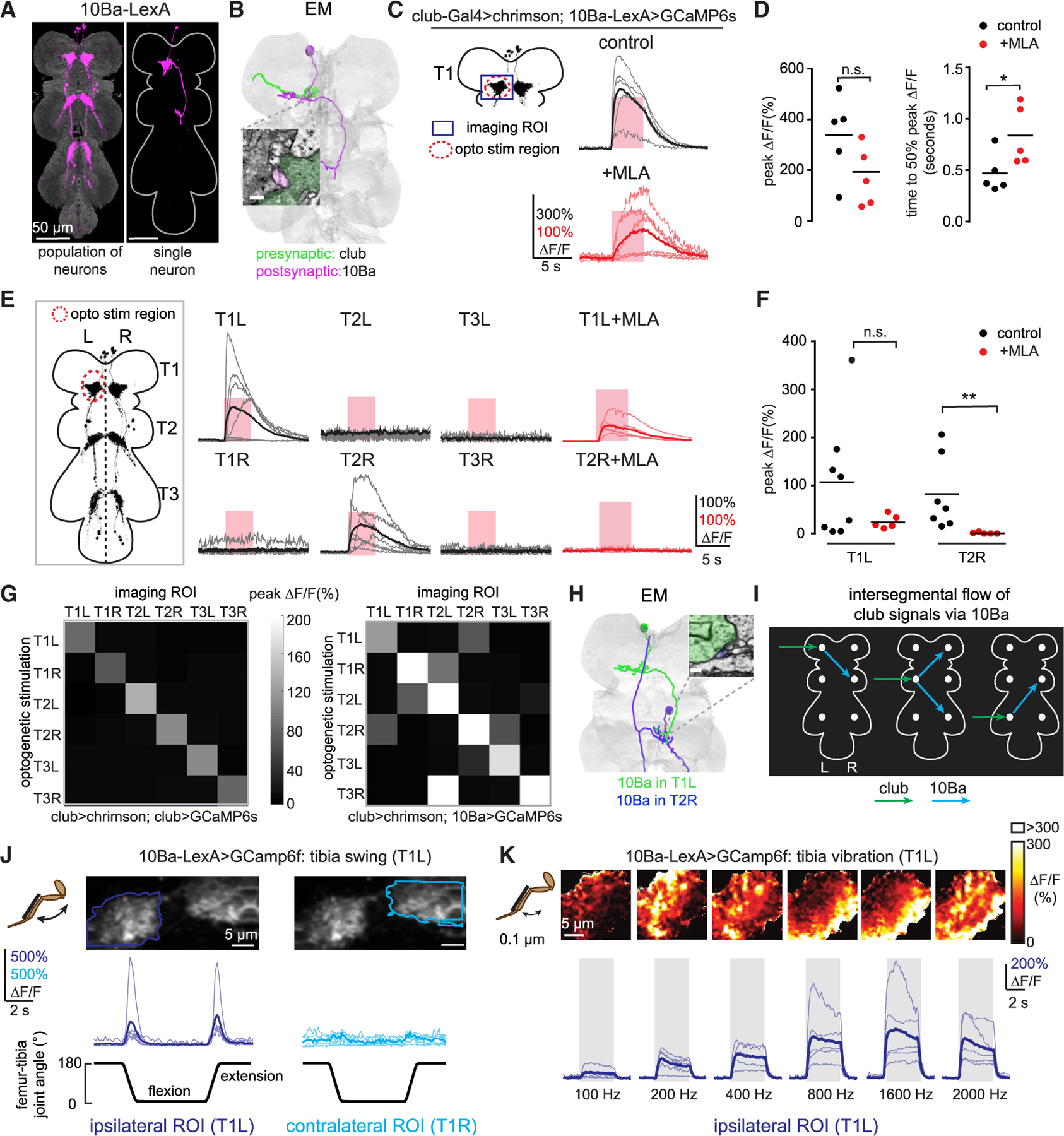
10Ba neurons integrate movement and vibration signals from club neurons across legs (A) Anatomy of 10Ba neurons. Magenta is GFP driven by *R13E04-LexA*; neuropil was stained by nc82. At right is a single neuron labeled by multi-color FLPout. Both images were aligned to a common VNC template. (B) Anatomical reconstruction from EM showing an example of a 10Ba neuron (magenta) that receives direct synaptic input from a club axon (green). The inset shows an example of a synapse between the two cells. Scale bar represents 200 nm. (C) Calcium responses of the 10Ba neurons to optogenetic stimulation of club neurons. Calcium responses of 10Ba neurons in the left prothoracic VNC (T1L) to stimulation of club neurons in the left foreleg are shown. MLA (1 μM) effectively blocked excitation from club neurons. The pink windows indicate stimulus duration (5 s; laser power = 0.28 mW/mm^2^). (D) Peak calcium responses (left) and time to 50% of the maximum calcium signal (right) across flies for the experiments shown in (C). Each dot represents data from a single fly; bars represent average peak calcium signals (left) or mean time to peak (right; control: n = 5; MLA: n = 5; *p < 0.05; Mann-Whitney test). (E) Calcium responses of 10Ba in all six neuromeres (T1L-T3R) to stimulation of club axons in T1L with or without MLA (1 μM). The pink windows indicate stimulus duration (5 s; laser power = 0.28 mW/mm^2^). (F) Same as in (D) but showing the quantification of the peak calcium responses shown in (E). Each dot represents data from a single fly (T1L: n = 8,5; T2R: n = 7,5; *p < 0.05; Mann-Whitney test). (G) Heatmaps of average peak calcium responses of club (left; n = 5 flies) and 10Ba (right; n = 6 flies) neurons in each neuropil to stimulation of the axons of club neurons in each leg. (H) Same as in (B) but showing a 10Ba neuron in T1 left (green) is connected to a 10Ba neuron in T2 right (blue) via EM reconstruction. Scale bar represents 200 nm. (I) Proposed diagram of signal flow from club axons to 10Ba neurons, based on data summarized in (G). White dots represent neurites of the 10Ba neurons in different neuromeres. (J) Calcium response in 10Ba neurons during tibia swing movement. 10Ba neurons respond phasically to bidirectional tibia movement (n = 6). (K) 10Ba neurons respond to tibia vibration. (Top) The majority of pixels had a ΔF/F value between 0% and 300%; outlier pixels with a value above 300% ΔF/F were set to white for visualization purposes. (Bottom) Calcium changes in 10Ba neurons during tibia vibration across different frequencies are shown. Thin lines are calcium signals from individual flies. The thicker line indicates the average across flies (n = 5).

We next switched our attention to 10Ba, the second candidate cell class whose anatomy suggests that it integrates club signals from multiple legs. A single 10Ba neuron with a cell body in T1 arborizes within one neuromere and then crosses the midline and arborizes in the contralateral T2 neuromere (Figure 3A).

A subset of 10Ba neurons also project to the brain, arborizing in the contralateral antennal mechanosensory and motor center (AMMC) (data not shown). Previous work showed that optogenetic activation of 10Ba neurons caused walking flies to pause,^20^ consistent with a role in detecting substrate vibration.^34^

We reconstructed 10Ba neurons in the EM volume and found dense synaptic inputs from club axons (Figure 3B; Table S2), confirming that club neurons are both functionally and anatomically presynaptic to 10Ba neurons. However, blocking acetylcholine receptors with MLA reduced but did not eliminate club-driven calcium signals in 10Ba neurons (Figures 3C and 3D). These data suggest that the connection between club and 10Ba neurons consists of mixed chemical and electrical signaling, which is consistent with previous work.^20^ Interestingly, we observed that the time to peak of the 10Ba calcium signals was significantly longer in the presence of MLA (Figure 3D), which suggests that calcium signals in 10Ba neurons mediated by chemical and electrical transmission may have distinct temporal dynamics.

The intersegmental projections of 10Ba neurons raise the possibility that these cells integrate signals from club neurons across different legs. To test this, we measured calcium responses of 10Ba neurons in each neuromere (T1L-T3R) while optogenetically stimulating club axons arising from each of the six leg nerves. When club axons from the left prothoracic (T1L) leg were optogenetically stimulated, we observed robust calcium signals in 10Ba processes of T1L and T2R, but not in other neuromeres (Figure 3E). Stimulating club axons in T1L also drove calcium activity in T2R (but not T2L) cell bodies (Figure S5). Applying MLA abolished calcium responses in T2R processes and cell bodies, but not in T1L (Figures 3E and 3F), suggesting a role for gap junctions in local, but not intersegmental, connectivity.

To test whether this connectivity pattern generalized to other VNC segments, we consecutively stimulated club axons from each leg while recording calcium signals from 10Ba neurons in all six neuromeres, resulting in a 6 × 6 functional connectivity map (Figure 3G). We observed intersegmental responses for all legs, though the pattern of information flow was different for each segment (Figure 3G, right). In contrast, stimulating and recording from the same club axons did not produce intersegmental responses (Figure 3G, left). These data suggest that 10Ba neurons integrate vibration signals directly from club axons within a segment and indirectly, via the intersegmental projections of 10Ba neurons, from other segments. This model is supported by our finding from EM reconstruction that 10Ba neurons from T1L and T2R are synaptically connected (Figure 3H; Table S2).

To compare these functional connectivity results to encoding of sensory stimuli, we recorded calcium signals in 10Ba neurons while moving the tibia passively at 180°/s. Unlike 9Ba, 10Ba neurons responded to ipsilateral, but not contralateral, tibia movement in the T1 segment (Figure 3J). Like club axons (Figure S1D), 10Ba neurons responded to tibia movement in both directions (Figure 3J), as well as high-frequency, low-amplitude vibration of the tibia (Figure 3K). The distribution of calcium signals shifted from lateral to medial as vibration frequency increased (Figure 3K), consistent with the topographic map of frequency previously observed in club axons.^19^ Curiously, this frequency map was not present in recordings from 9Ba neurons (Figure 2H).

In summary, both 9Ba and 10Ba neurons encode bidirectional movements and vibration signals by integrating club axons across multiple legs. The key difference between these cell classes is that 9Ba neurons mediate bilateral inhibition within a VNC segment, whereas 10Ba are excitatory neurons that integrate club inputs across contralateral VNC segments (Figure 3I). This convergence supports our hypothesis that club pathways play a role in detecting external vibration stimuli, which are likely to be synchronized across legs.

### 13Bb neurons integrate position and directional movement signals

Another interesting result of our functional connectivity screen was that some second-order neurons integrate proprioceptive signals across multiple FeCO subtypes. Specifically, we identified two candidate cell types (13Bb and 8Aa) downstream of both claw and hook axons. We selected 13Bb neurons for further analysis because clean genetic driver lines exist for this cell class.

**Figure 4.**
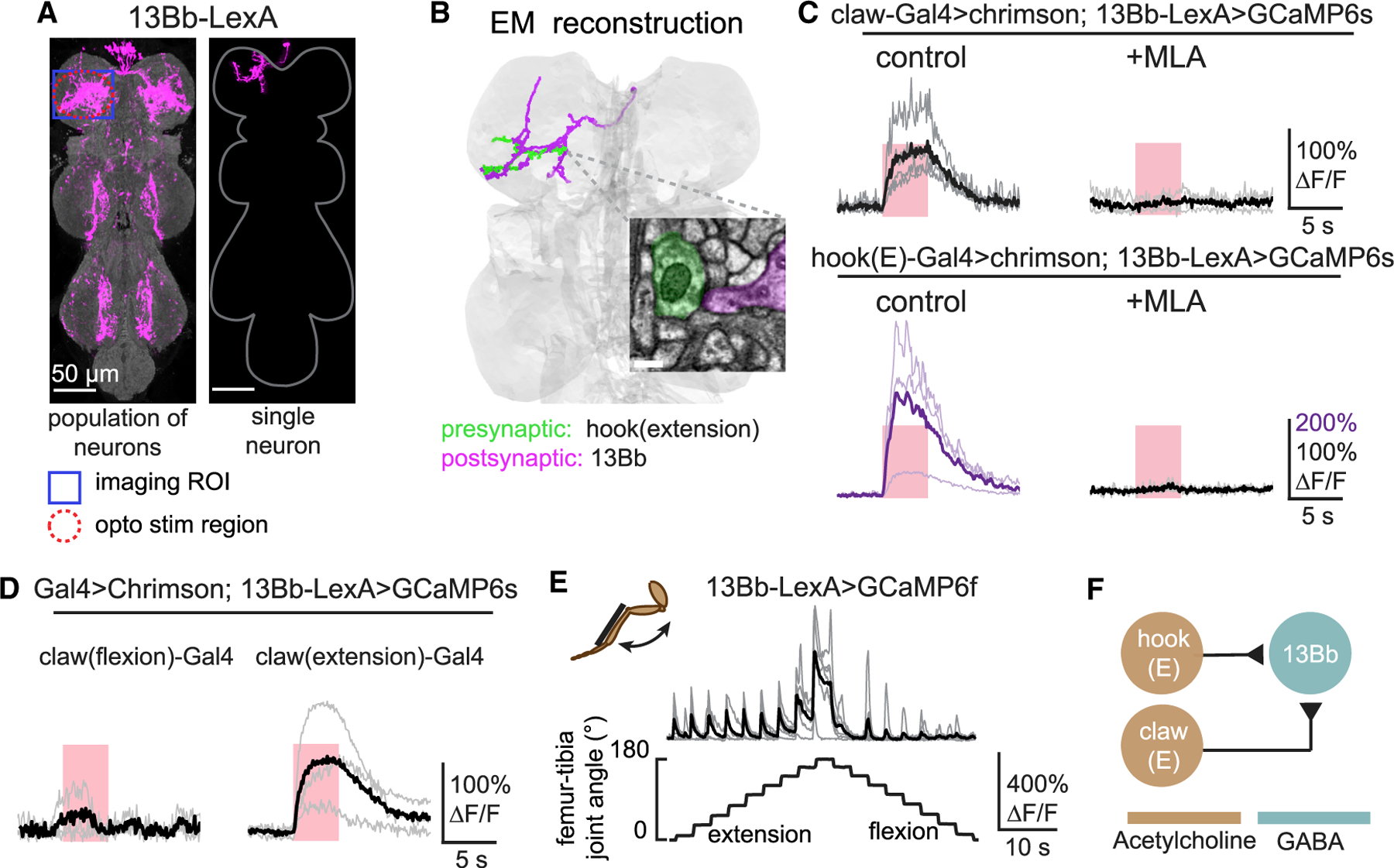
13Bb neurons integrate position and directional movement signals from claw and hook (extension) neurons (A) Anatomy of 13Bb neurons. Population (left) and single neuron (right) anatomy of 13Bb neurons are shown. GFP (magenta) was driven by *VT006903-LexA*. The VNC neuropil was stained against nc82 (gray). Both images were aligned to a common VNC template. (B) Anatomical reconstruction using EM showing an example of a 13Bb neuron (magenta) that receives direct synaptic input from a hook (extension) axon (green). The inset shows an example of a synapse between the two cells. Scale bar is 200 nm. (C) Calcium responses of 13Bb neurons to optogenetic stimulation of claw and hook (extension) neurons. (Left) Calcium responses of 13Bb neurons in the left prothoracic VNC to optogenetic stimulation of claw axons from the left foreleg (T1L) are shown. (Right) MLA (1 μM) blocked excitation produced by claw neuron activation. The pink windows indicate stimulus duration (5 s; laser power = 0.28 mW/mm^2^). Control: n = 5 and MLA: n = 4, respectively. (Bottom) Calcium responses of 13Bb neurons to optogenetic stimulation of hook (extension) axons are shown. (D) Calcium responses of 13Bb to optogenetic stimulation of claw-flexion (n = 3) and claw-extension axons (n = 4). (E) 13Bb neurons respond to tibia extension. Calcium changes of 13Bb neurons during tibia movement (n = 6) are shown. (F) Proposed diagram of sensory integration by 13Bb neurons, which receive input from claw-extension and hook (extension) neurons.

We reconstructed the anatomy of 13Bb cells from the EM volume and found direct synaptic inputs from hook (extension) axons (Figure 4B; Table S2). We did not find any synapses between 13Bb and claw neurons, probably because only 2 claw axons were fully reconstructed; however, we cannot rule out the possibility that claw axons are connected to 13Bb neurons indirectly. Calcium responses in 13Bb neurons were abolished when we blocked acetylcholine receptors with MLA (Figure 4C), suggesting that the synaptic input from both FeCO subtypes relies on chemical synaptic transmission.

We next sought to understand the convergence of position and movement signals within 13Bb neurons. Our functional connectivity screen revealed that 13Bb neurons respond to activation of claw axons, but the driver line we used to activate these neurons labeled both flexion- and extension-tuned cells. We therefore created Split-Gal4 lines that separately label claw neurons encoding tibia flexion (<90°) and extension (>90°; Figure S6) and repeated functional connectivity experiments with these sparser lines. 13Bb neurons specifically increased calcium activity in response to optogenetic stimulation of extension-tuned claw neurons but did not respond to flexion-tuned claw neurons (Figure 4D). Calcium responses during passive tibia movements were consistent with convergent input from extension-tuned claw and hook neurons: we observed phasic responses during tibia extension across the full range, which we attribute to hook input, and tonic responses above 140°, which we attribute to claw input. The amplitudes of the tonic and phasic calcium signals were similar, such that the 13Bb calcium signals peaked during extension movements when the tibia was already extended (Figure 4E).

In summary, GABAergic 13Bb neurons integrate excitatory input from extension-tuned claw and hook neurons to encode joint movement within a specific angular range (Figure 4F). Integration of direction and position signals could be beneficial for dynamic tuning of resistance reflexes that maintain body posture and protect joints from hyperextension.

### Inhibition gates calcium dynamics of central proprioceptive pathways

**Figure 5.**
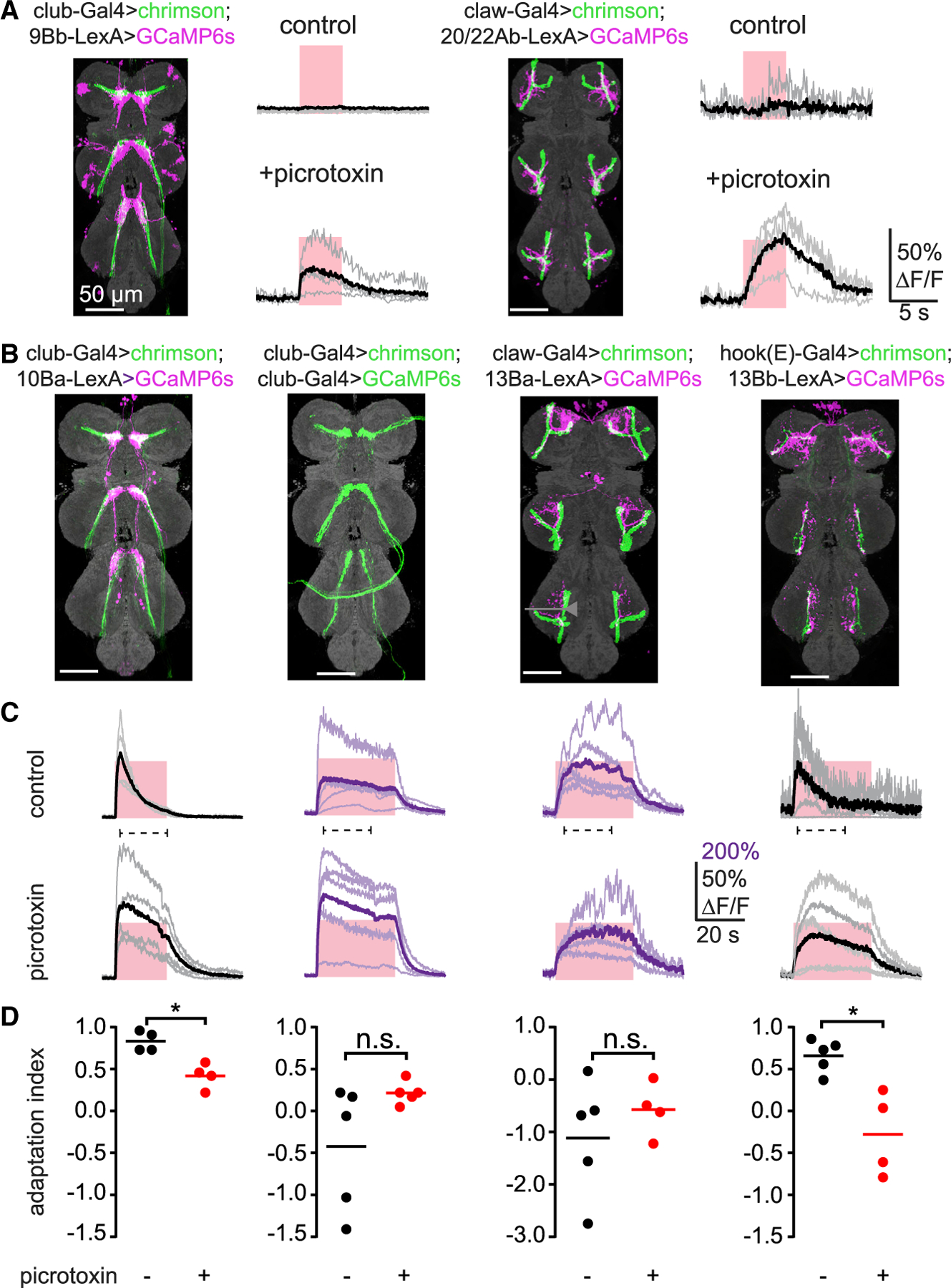
Multiple roles for inhibition in functional connectivity between first- and second-order proprioceptive neurons (A) Inhibition gates connectivity between leg proprioceptors and VNC neurons. Left: responses of 9Bb neurons to optogenetic stimulation of club neurons were revealed after application of picrotoxin (10 μM). Right: similar results for claw and 20/22Ab neurons are shown. The pink windows indicate stimulus duration (5 s; laser power = 0.28 mW/mm^2^). (B) Anatomy of the axons of FeCO subtypes (green) and their downstream targets (magenta). VNC neuropil was stained using nc82 (gray). (C) Calcium responses of second-order neurons in the left prothoracic VNC to optogenetic stimulation of the indicated sensory neurons (top). Picrotoxin (10 μM) reduced response adaptation in 10Ba and 13Bb neurons. The pink windows indicate the optogenetic stimulus duration (20 s for 10Ba neurons and 30 s for others; the laser power was 0.28mW/mm^2^). The dashed line under each trace indicates the window used to calculate the adaptation index below. (D) Quantification of calcium signal adaptation from data in (C). Adaptation index was calculated as 1 − F_offset_/F_peak_. 1 indicates complete adaptation, 0 indicates no adaptation, and negative values indicate an increase of the calcium signal over time. Each dot represents data from a single fly. Bars indicate the average (*p < 0.05; Mann-Whitney test).

Our screen (Figure 1) identified several cell classes with dendrites in close proximity to FeCO axons, but which did not respond to optogenetic activation of proprioceptors. We wondered whether their connectivity was masked by inhibition, as has been demonstrated in second-order neurons that process tactile signals from the leg.^11^ We repeated functional connectivity experiments with these cell classes while blocking GABA_a_ receptors with picrotoxin (10 μM). Of the 8 VNC cell classes we tested, two (9Bb and 20/22Ab) had measurable calcium signals only in the presence of picrotoxin (Figure 5A). These connections were specific, meaning that 9Aγ neurons responded only to activation of club neurons and 20/22Ab neurons responded only to activation of claw neurons. We therefore hypothesize that removing inhibition unmasks latent excitatory input from leg proprioceptors.

In other cell classes, we found that GABAergic inhibition sculpted the dynamics of the calcium response. For example, GCaMP signals recorded from 10Ba neurons adapted during prolonged optogenetic stimulation (20 s) of club neurons (Figure 5C). In contrast, GCaMP signals in 13Ba neurons remained elevated during optogenetic stimulation of claw neurons over a period of 30 s (Figure 5C). This adaptation was not due to decay of optogenetically evoked activity in the proprioceptor axons. Rather, it appears that inhibition, likely mediated by GABA_a_ or GluCl receptors,^35^ contributes to the adaptation of 10Ba calcium signals during prolonged stimulation. We observed a similar phenomenon for 13Bb neurons during optogenetic stimulation of hook (extension) neurons (Figure 5C). Overall, these results show that adaptation mediated by GABAergic or glutamatergic inhibition gates the activity and sculpts the dynamics of second-order proprioceptive neurons.

## DISCUSSION

In this study, we report the anatomical structure and functional organization of second-order circuits for leg proprioception in *Drosophila*. Due to the lack of clear hierarchical structure within the VNC leg neuropil, it has been challenging to infer the flow of proprioceptive sensory signals with existing tools. We therefore generated genetic driver lines that label specific subtypes of leg proprioceptors and classified candidate second-order neurons based on hemilineage identity. We used optogenetics and calcium imaging to map the functional connectivity between leg proprioceptors and second-order neurons, followed by EM reconstruction to validate synaptic connectivity and *in vivo* calcium imaging to understand the function of second-order neurons during leg movement. We then used spatially targeted and subtypespecific optogenetic stimulation to analyze integration of FeCO signals within a subset of second-order neuron classes.

**Figure 6.**
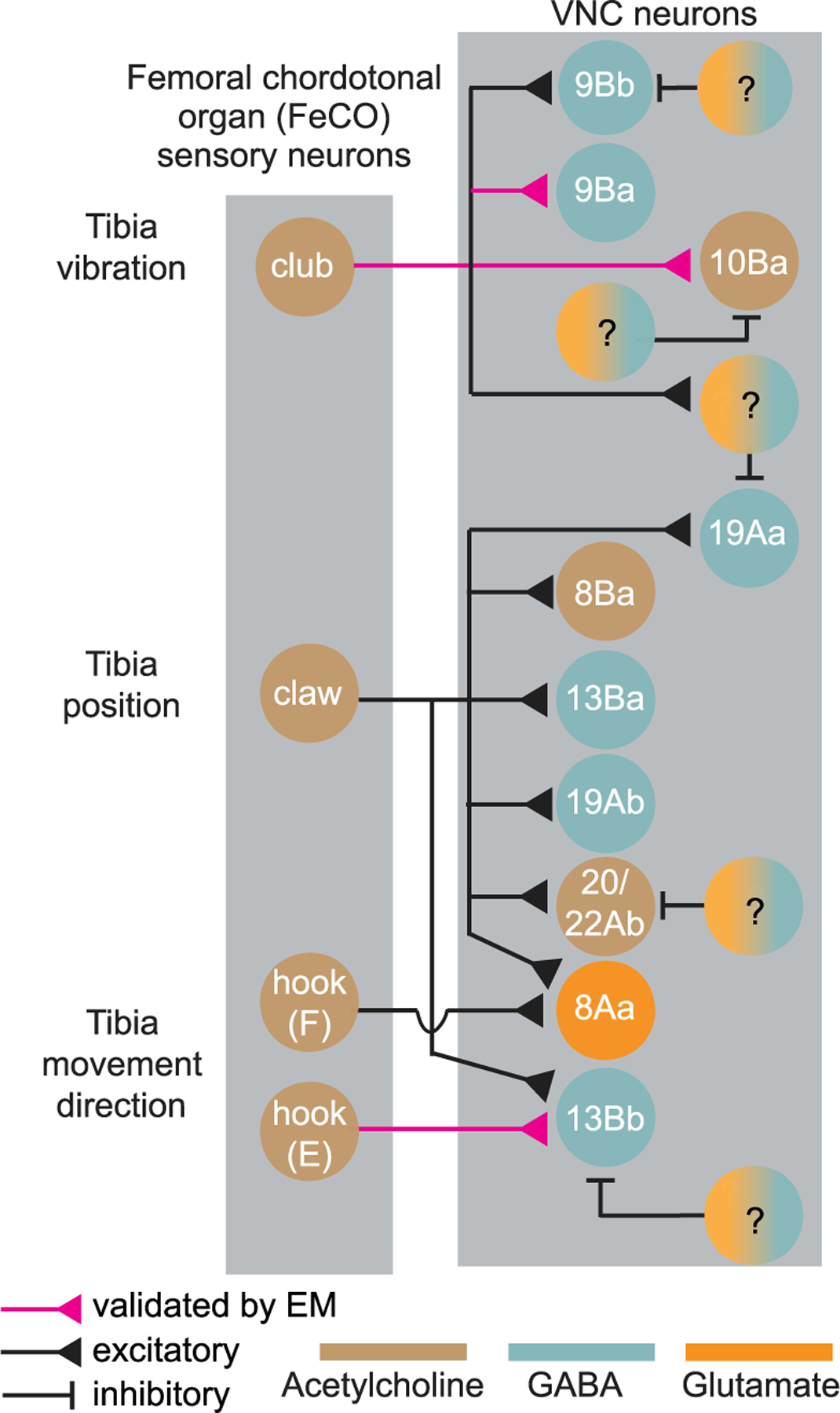
Summary diagram of circuits processing leg proprioceptive signals from the *Drosophila* FeCO, based on experiments in this study Question marks indicate putative inhibitory neurons of unknown identity.

Overall, this work reveals the logic of sensory integration in second-order proprioceptive circuits: some populations of second-order neurons integrate tibia vibration signals across pairs of legs, suggesting a role for detection of external substrate vibration. Signals for leg joint position and directional movement converge in other second-order neurons, revealing pathways for local feedback control of leg posture. We anticipate that this functional wiring diagram (Figure 6) will also help guide the interpretation of anatomical wiring diagrams determined through EM reconstruction of VNC circuits.

### Connectivity motifs within second-order proprioceptive circuits

Proprioceptors in the *Drosophila* FeCO can be classified into three subtypes: club neurons encode bidirectional tibia movement and vibration frequency; claw neurons encode tibia position (flexion or extension); and hook neurons encode the direction of tibia movement.^19^ Our results show the existence of two distinct central pathways for processing signals from club vs. claw and hook neurons (Figure 6). We propose that neurons downstream of the club mediate sensing of small mechanical vibrations in the external environment, whereas neurons downstream of the claw and hook provide proprioceptive feedback to motor circuits for controlling the posture and movement of the legs. This division of central pathways for external and internal sensing may be a common motif across limbed animals. Work in a variety of species, including a recent study in mice,^36^ has found that many animals can detect low-amplitude, high-frequency, substrate-borne vibrations.^37^ Flies may use vibration sensing to monitor acoustic signals in the environment, such as during courtship behavior,^38^ or to detect approaching threats.

The distinct anatomical organization of neurons downstream of the club vs claw and hook also supports a segregation of vibration sensing and motor control feedback pathways. 9Ba and 10Ba neurons arborize in the mVAC (Figure 1E), a common target of descending neuron axons.^39^ In contrast, 13Bb arborize in the IntNp (Figure 1E), which contains the dendritic branches of the leg motor neurons and premotor interneurons. Based on these differences, we hypothesize that vibration-sensing neurons interact with ascending and descending signals to and from the brain, whereas neurons downstream of hook and claw axons contribute to local motor control through direct or indirect connections to motor neurons. Leg motor neurons receive position- and movement-tuned proprioceptive input, consistent with feedback from claw and hook neurons.^40^ Additional connectomic reconstruction is needed to determine which second-order neurons mediate these feedback connections, but 13Bb neurons are promising pre-motor candidates.

We found that VNC neurons postsynaptic to claw and hook axons receive only local input, from individual legs. In contrast, second-order neurons postsynaptic to club axons integrate signals across multiple legs. For example, GABAergic 9Bb neurons pool information from left and right legs in a single VNC segment (Figure 2), whereas cholinergic 10Ba neurons receive convergent input from left and right legs across different segments (Figure 3). Integrating club input across legs may improve detection of external vibration signals, while proprioceptive signals from the claw and hook may be initially processed in parallel to support postural control of individual legs. Bilateral integration also occurs in second-order auditory circuits downstream of the *Drosophila* Johnston’s organ: mechanosensory signals from the two antennae are processed in parallel by second-order neurons in the AMMC but then converge in third-order neurons in the wedge.^41^

Although second-order neurons in the vibration pathway integrate club signals across legs, multiple classes of second-order neurons in the motor pathway (13Bb and 8Aa) integrate signals across different FeCO subtypes from the same leg. Using new genetic driver lines that subdivide claw neurons into extension- and flexion-tuned subtypes, we found that extension-tuned claw and hook neurons converge on 13Bb neurons. We hypothesize that these cells mediate resistance reflexes that stabilize tibia position in response to external perturbations. Prior work in the stick insect has shown that tibia resistance reflexes rely on position and directional movement signals from the FeCO.^42^ In support of this hypothesis, another class of neurons in the 13B hemilineage, 13Ba, also encode tibia extension and drive tibia flexion when optogenetically activated.^20^

This functional connectivity map reveals interesting parallels with sensorimotor circuitry in the larval *Drosophila* VNC. Although fly larvae do not have legs, they do possess body wall proprioceptors (class I sensory neurons) and use chordotonal neurons to sense external vibrations in a manner analogous to club neurons in the adult FeCO. As in the adult, larval neurons belonging to lineage 9 (basin neurons)^43,44^ and lineage 8 (eve lateral interneurons)^45^ integrate signals from chordotonal sensory neurons and proprioceptors. These examples suggest that some lineage connectivity motifs are likely conserved across the larval and adult nervous systems, which are already known to possess molecular and general anatomical similarities.^28^

### Inhibition and temporal dynamics

Our results identify a prominent role for inhibition in central processing of proprioceptive information from the FeCO. Of the eight second-order cell classes we identified in our screen, six are putative inhibitory neurons (i.e., release GABA or glutamate). In other sensory circuits, local inhibitory processing contributes to sharpening spatial and temporal dynamics^46^ as well as reducing sensory noise through crossover inhibition.^47,48^ By pharmacologically blocking GABA_a_ and GluCl receptors, we identified a role for inhibition in controlling adaptation within second-order neurons (e.g., 10Ba and 13Bb neurons; Figure 5C). In other cases (20/22Ab or 9Ba; Figure 5A), inhibition was strong enough to completely mask proprioceptive inputs from FeCO axons. We hypothesize that this inhibition may be tuned in certain behavioral contexts, for example, during active movements, to gate the flow of proprioceptive feedback signals in a context-dependent manner.

Synaptic transmission in *Drosophila* can be mediated by chemical synapses, which can be visualized with EM, or electrical gap junctions, which are not typically identifiable at the resolution of current EM volumes. FeCO neurons release acetylcholine but also express gap junction proteins (*shakB*; data not shown). We therefore used pharmacology to test for the presence of gap junctions between sensory and central neurons. MLA, an effective antagonist of nicotinic acetylcholine receptors in *Drosophila*,^11^ eliminated functional connectivity between club and 9Bb neurons but only reduced functional connectivity between club and 10Ba neurons. We observed similar results downstream of the claw: MLA blocked functional connectivity between claw and 13Bb neurons but only reduced functional connectivity between claw and 13Ba neurons (data not shown). These results suggest that second-order proprioceptive circuits receive mixed chemical and electrical input from FeCO neurons. More work is needed to confirm these observations and to investigate the functional significance of why pathways might use one means of signal transmission over the other. One hypothesis is that chemical synapses exhibit adaptation (e.g., synaptic depression), whereas electrical synapses may be more advantageous for sustained synaptic transmission.^49^ Each may provide different advantages for pathways that control behavior on a variety of timescales, from slow postural reflexes to rapid escape behaviors.

### Comparison to central mechanosensory processing in other species

Central processing of sensory signals from the FeCO has been previously studied in other insects, especially the locust^2^ and stick insect.^3^ In both species, second-order interneurons encode combinations of tibia movement and position^15^ and also integrate multimodal signals from different proprioceptive organs.^50,51^ Vibration signals detected by the FeCO appear to be processed by largely segregated populations of VNC interneurons.^15,52^ However, these conclusions were based on mapping of sensory receptive fields, and it was not previously possible to identify specific sources of sensory input, as we do in this study.

Overall, comparison of our functional connectivity results in *Drosophila* with the prior work in other insect species suggests general evolutionary conservation of VNC circuits for leg proprioception and motor control. Although it is currently difficult to identify homologous cell types across insect species, future efforts could leverage conserved developmental programs: the organization of neuroblasts that give rise to the VNC is similar across insect species separated by 350 Ma of evolution.^28^ This is an important advantage of using developmental lineages to define VNC cell classes—locusts and stick insects also possess 9A, 10B, and 13B neurons, which could someday be identified based on molecular markers of lineage identity.

### Complementary strengths of functional and structural connectivity

The functional connectivity approach that we employed in this study has both benefits and drawbacks. On the positive side, it allowed us to screen a large connectivity matrix of genetically identified sensory and central neurons. Compared to other methods for anatomical mapping (e.g., EM), the use of optogenetics and calcium imaging allowed us to measure connection strength and dynamics across multiple individuals. We found that second-order VNC neurons varied significantly in their functional connectivity strength and temporal dynamics (Figures 1G and 1H). We also observed 5-fold differences in peak calcium signals in response to optogenetic stimulation with the same light intensity (Figure 1G). This range could be due to differences in GCaMP expression or intracellular calcium buffering, but could also reflect differences in synaptic strength between preand postsynaptic partners.

One limitation of functional connectivity is that it is not possible to measure all possible combinations of pre- and postsynaptic partners. For example, a previous study^20^ provided evidence that 9Aa neurons receive input from hook and club neurons, which we did not observe in our screen (Figure 1C). This discrepancy could be due to the fact that the driver lines we used do not label the specific subset of hook and club cells presynaptic to 9Aa neurons. Alternatively, it may be due to differences in signal transmission driven by optogenetic stimulation versus natural tibia movements, as was the case for 9Bb neurons (Figure 5A).

Functional connectivity mapping also cannot resolve whether inputs are direct, due to the slow kinetics of GCaMP6. We therefore used sparse, targeted EM tracing to validate some of the functional connections we identified between FeCO and VNC neurons. A more detailed comparison of functional and anatomical connectivity will require dense, comprehensive reconstruction of the VNC neuropil. Automated reconstruction and manual proofreading have recently led to draft wiring diagrams of neural circuits in the adult *Drosophila* central brain.^53^ Similar approaches to reconstruct the VNC connectome are in progress.^17^

## STAR★METHODS

### RESOURCE AVAILABILITY

#### Lead contact

Further information and request for resources and reagents should be directed to the Lead Contact, John C. Tuthill (tuthill@uw.edu).

#### Material availability

Fly lines generated in this study are available without restrictions from the Lead Contact, John C. Tuthill (tuthill@uw.edu).

#### Data and code availability

Data will be made available on Dryad (https://doi.org/10.5061/dryad.rfj6q57bm).

### EXPERIMENTAL MODEL AND SUBJECT DETAILS

#### Fly stocks

*Drosophila* were raised on cornmeal agar food on a 12h dark/12h light cycle at 25°C. Female flies, 4–8 days post eclosion, were used for all calcium imaging experiments. For functional connectivity experiments, adult flies carrying the Chrimson transgene were placed on cornmeal agar with all-trans-retinal (0.2 mM, dissolved in 95% EtOH, Sigma-Aldrich) for 2–3 days prior to the experiment. Vials were wrapped in aluminum foil to reduce unnecessary optogenetic activation. Fly stocks used in this study are described and listed in Table S3.

### METHOD DETAILS

#### Creation of Split-GAL4 lines for targeting proprioceptors in fly leg

GAL4 images from the Rubin and Dickson collections^23,24^ were visually screened for lines labeling axons from proprioceptors that project to the VNC. For each cell type, a color depth MIP mask search^54^ was performed to find other GAL4 lines with expression in similar cells. Split-GAL4 AD and DBD hemi-drivers^24,55^ for these lines were crossed in several different combinations to identify intersections that targeted the cell type of interest but with minimal expression elsewhere. Sensory tuning properties of FeCO subclass neurons labeled by these Split-Gal4 lines were further characterized using *in vivo* calcium imaging, described below.

#### Immunohistochemistry and anatomy

For confocal imaging of the FeCO neuron axons driven by each Split-Gal4 line in the VNC (Figures S1 and S6), we crossed flies carrying the Split-Gal4 driver to flies carrying 20xUAS-IVS-mCD8::GFP or 20xUAS-Chrimson::mVenus (attp18) and dissected the brain and VNC of the resulting progeny in cold Schneider’s Insect Medium (S2). The tissues were first fixed in 2% paraformaldehyde (PFA) PBS solution for 55 min followed by rinsing in PBS with 0.5% Triton X-100 (PBT) four times. The brain and VNC were blocked in solution (5% normal goat serum in PBT) for 90 min, then incubated with a solution of primary antibody (rabbit anti-GFP 1:1,000 concentration; mouse nc82 for neuropil staining; 1:30 concentration) in blocking solution for 4 hr, followed by washing tissues in PBT three times. Tissues were incubated with a solution of secondary antibody (anti-rabbit-Alexa 488 1:400 concentration; anti-mouse-Alexa 633 1:800 concentration) dissolved in blocking solution for 4 hr followed by three times washing with PBT before DPX mounting. The whole procedure was performed at room temperature.

For stochastic labeling of individual neurons in the VNC, we crossed flies carrying the multicolor FlpOut cassettes and Flp recombinase drivers to flies carrying different Split-Gal4 and LexA drivers and dissected out the VNCs of resulting progeny. For temperature induced expression of Flp, we placed adult flies at 1–3 days old in a plastic tube and incubated them in a 37°C water bath for 15 min. We dissected the VNC four days after the Flp induction and followed the procedure described in Nern et al.^56^ to detect HA (using anti-HA-rabbit antibody and anti-Rabbit-Alexa 594 secondary antibody), V5 (using DyLight 549-conjugated anti-V5), and FLAG (using anti-FLAG-rat antibody and anti-Rat-Alexa 647 secondary antibody) labels expressed due to Flp induction in individual neurons.

For trans-Tango experiments, flies were aged at 18 °C for 15–40 days before dissection. We follow the standard staining protocol, described above. The antibody concentrations used in those experiments were as follows: primary rabbit anti-GFP (1:1000), primary rat anti-HA (1:100), primary mouse anti-Bruchpilot (1:20), secondary Alexa Fluor 488 goat anti-rabbit (1:500), secondary Alexa Fluor 555 goat anti-rat (1:800), and secondary Alexa Fluor 647 goat anti-mouse (1:500).

Images were acquired on Zeiss LSM 710 or 800 confocal microscopies with 20x or 63x objectives. We used Fiji^57^ to generate maximum intensity projections of the expression of driver lines as well as anatomy of single neurons.

#### Fly preparation for two-photon calcium imaging

For functional connectivity experiments, adult female flies were anesthetized on ice and then glued to a Petri dish ventral side up using UV-cured glue (Kemxert 300). To eliminate spontaneous activity due to fly leg movement, we amputated the legs at the coxa joint. After immersing the fly in extracellular fly saline (103mM NaCl, 3mM KCl, 2mM CaCl_2_, 4mM MgCl_2_, 26mM NaHCO_3_, 1mM NaH_2_PO_4_, 8mM trehalose, 10mM glucose, 5mM TES, pH 7.1, osmolality adjusted to 270–275 mOsm, bubbled with 95% O_2_ / 5% CO_2_), we removed the cuticle above the T1 segment of the VNC and took out the digestive tract to reduce the movement of the VNC. Recordings were performed at room temperature.

For calcium imaging during controlled leg movements, we used a fly holder previously described by Mamiya et al.^19^ Flies were anesthetized on ice and then positioned ventral side up, with the head glued to the upper side of the fly holder using UV-cured glue (Kemxert 300). We glued the ventral side of the thorax on the bottom side of the holder and glued down the femur of the right T1 leg so that we could control the femur-tibia joint angle by moving the tibia. When gluing the femur, we held it at a position where the movement of the tibia during the rotation of the femur-tibia joint was parallel to the plane of the fly holder. To eliminate mechanical interference, we also glued down the other legs. We pushed the abdomen to the left side and glued it at that position, so that the abdomen did not block tibia flexion. To position the tibia using the magnetic control system described below, we cut a small piece of insect pin (length ~1.0 mm, 0.1 mm diameter; Living Systems Instrumentation) and glued it onto the tibia and the tarsus of the right T1 leg. To enhance contrast and improve tracking of the tibia/pin position, we painted the pin with black India ink (Super Black, Speedball Art Products). After immersing the ventral side of the preparation in extracellular fly saline, we removed the cuticle above the T1 segment of the VNC and took out the digestive tract to reduce the movements of the VNC. We also removed fat bodies and larger trachea to improve access to the leg neuropil. Fly saline contained: 103mM NaCl, 3mM KCl, 2mM CaCl_2_, 4mM MgCl_2_, 26mM NaHCO_3_, 1mM NaH_2_PO_4_, 8mM trehalose, 10mM glucose, 5mM TES, pH 7.1, osmolality adjusted to 270–275 mOsm. Recordings were performed at room temperature.

#### Image acquisition using a two-photon excitation microscope

For functional connectivity experiments, images were acquired using a two-photon microscope (custom-made at Janelia by Dan Flickinger and colleagues, with a Nikon Apo LWD 25 × NA1.1 water immersion objective). The standard imaging mode was a 512 × 512 image at 2.5 frames/s, and a ~353 μm × ~353 μm field of view (~0.69 μm × ~0.69 μm / pixel). The sample was imaged using a near-infrared laser (920nm, Spectra Physics, Insight DeepSee) that produced minimal activation of Chrimson at our typical imaging power (4–10 mW). Chrimson was activated by 590nm light (Thorlabs M590L3-C1) presented through the objective. Photo-activation light was delivered in a pulse train that consisted of six 5 s pulses (within each 5 s pulse: square-wave modulation at 50 Hz, 30 s inter-pulse interval). The light intensity increased for each of the six pulses (0.02, 0.04, 0.12, 0.28, 0.37, 0.68 mW/mm^2^). For targeted stimulation (e.g., Figures 2C and 3E), illumination was spatially modulated using a DMD (Digital Micromirror Device, Texas Instruments, DLP LightCrafter v2.0), and restricted to a specific stimulation region.

For calcium imaging during controlled leg movements, we used a modified version of a custom two-photon microscope, previously described in detail^58^. For the excitation source, we used a mode-locked Ti/sapphire laser (Mira 900-F, Coherent) set at 930 nm and adjusted the laser power using a neutral density filter to keep the power at the back aperture of the objective (40x, 0.8 NA, 2.0 mm wd; Nikon Instruments) below ~25 mW during the experiment. We controlled the galvo laser scanning mirrors and the image acquisition using ScanImage software (version 5.2) within MATLAB (MathWorks). To detect fluorescence, we used an ET510/80M (Chroma Technology Corporation) emission filter (GCaMP6f or GCaMP6s) and a 630 AF50/25R (Omega optical) emission filter (tdTomato) and GaAsP photomultiplier tubes (H7422P-40 modified version without cooling; Hamamatsu Photonics). We acquired images (256 × 120 pixels or 128 × 240 pixels) at 8.01 Hz. At the end of the experiment, we acquired a z stack of the labeled neurons to confirm the recording location.

#### Image processing and calculating ΔF/F

We performed all calcium image processing and analyses using scripts written in MATLAB (MathWorks). After acquiring the images for a trial, we first applied a Gaussian filter (size 5×5 pixel, Ʃ = 3) and intensity threshold to minimize background noise. For calculating the GCaMP6 fluorescence change relative to the baseline (ΔF/F), we used the lowest average fluorescence level in a 10-frame window as the baseline fluorescence during that trial. For cases in which calcium signals were reduced relative to baseline (e.g., 19Aa neurons), we used the average fluorescence level in a 10-frame window at the beginning of each trial as the baseline. Because not all flies co-expressed tdTomato, we did perform image registration to correct for sample movement. From those flies that did co-express tdTomato, we observed that movement of the VNC was negligible.

We defined three parameters to analyze the temporal dynamics of calcium signals, as shown in Figure 1G: peak ΔF/F during the stimulation window, the time after stimulation at which the ΔF/F reaches 50% of the peak value (Figure 1I), and the half-decay time after the peak ΔF/F is reached (Figure 1J). For quantification of adaptation in Figure 5D, we calculated an adaptation index as 1 − F_offset_/F_peak_, where F_peak_ indicates the peak ΔF/F, and F_offset_ is ΔF/F 19 s after the stimulus onset (where the stimulation offset typically occurs in club/10Ba neurons). An adaptation index of 1 would indicate 100% decay to baseline, while an index of 0 would indicate no adaptation. Negative index values indicate an increase in the calcium signal over time.

#### Moving the tibia using a magnetic control system

We used a previously described magnetic control system^19^ to manipulate the femur/tibia joint angle. To move the tibia/pin to different positions, we attached a rare earth magnet (1 cm height × 5 mm diameter column) to a steel post (M3×20 mm flat head machine screw) and controlled its position using a programmable servo motor (SilverMax QCI-X23C-1; Max speed 533,333°/s, Max acceleration 83,333.33°/s^2^, Position resolution 0.045°; QuickSilver Controls). To move the magnet in a circular trajectory centered at the femur-tibia joint, we placed the motor on a micromanipulator (MP-285, Sutter Instruments) and adjusted its position while visually inspecting the movement of the magnet and the tibia using the tibia tracking camera described below. For each trial, we controlled the speed and the position of the servo motor using QuickControl software (QuickSilver Controls). During all trials, we tracked the tibia position (as described below) to confirm the tibia movement during each trial. Because it was difficult to fully flex the femur-tibia joint without the tibia/pin and the magnet colliding with the abdomen, we only flexed the joint up to ~18°. We set the acceleration of the motor to 72,000°/s^2^ for all ramp and hold and swing movements. Movements of the tibia during each trial varied slightly due to differences in the length of the magnetic pin and the positioning of the tibia and motor.

#### Tracking the femur-tibia joint angle during imaging experiments

To track the position of the tibia, we illuminated the tibia/pin with an 850 nm IR LED (M850F2, ThorLabs) and recorded video using an IR sensitive high-speed video camera (Basler Ace A800–510um, Basler AG) with a 1.0x InfiniStix lens (94 mm wd, Infinity). The camera was equipped with a 900 nm short pass filter (Edmund Optics) to filter out the two-photon laser light. To synchronize the tibia movement with the recorded cell activity, the camera exposure signal and the position of the galvo laser scanning mirrors were acquired at 20 kHz. To track the tibia angle, we identified the position of the painted tibia/pin against the contrasting background by thresholding the image. We then approximated the orientation of the leg as the long axis of an ellipse with the same normalized second central moments as the thresholded image.

#### Vibrating the tibia using a piezoelectric crystal

To vibrate the tibia at high frequencies, we moved the magnet using a piezoelectric crystal (PA3JEW, Max displacement 1.8 μm; ThorLabs). To control the movement of the piezo, we generated sine waves of different frequencies in MATLAB (sampling frequency 10 kHz) and sent them to the piezo through a single channel open-loop piezo controller (Thorlabs). Piezo-induced tibia movements during the calcium imaging prep were calibrated as described^19^. For each stimulus, we presented 4 s of vibration 2 times with an inter-stimulus interval of 8 s. We averaged the responses within each fly before averaging across flies.

#### Pharmacology

Drugs were added to the bath with a micropipette. Picrotoxin (Sigma-Aldrich) was prepared as a concentrated stock solution in aqueous NaCl (140 mM), and methyllycaconitine citrate (MLA, Sigma-Aldrich) was prepared as a stock solution in water. Each drug was further diluted in saline for experiments for a final concentration of 1 μM (MLA), or 10 μM (picrotoxin). The VNC was incubated in the drug for 5 min, with the perfusion system off, before starting the experiment, which typically lasted ~30 min.

#### EM reconstruction

A TEM volume of the adult female VNC^17^ was used for reconstruction of neurons and their synaptic connectivity. FeCO axons were traced manually using CATMAID, a collaborative manual tracing environment^59^. Second-order neurons were identified and matched to light-level data based on common hemilineage characteristics: primary neurite fasiculation, dendritic arborizations, and axon projections^60^. Neurons were segmented in EM (methods in preparation), proofread in Neuroglancer (https://github.com/google/neuroglancer), skeletonized, and imported to CATMAID, where further proofreading was conducted. Postsynaptic sites on VNC neurons were identified by the presence of a dark post-synaptic density and a corresponding T-bar on the presynaptic cell, consistent with standards in the field^61^. Beginning with a synapse on each VNC neuron, sensory neurons were traced from the synapse back to the incoming axon such that they could be identified. We focused on identifying a minimal basis for connectivity between first and second-order neurons, due to ongoing efforts to automatically segment the entire VNC volume. See Table S2 for synapse counts.

### QUANTIFICATION AND STATISTICAL ANALYSIS

For functional connectivity results in Figures 1, 2, 3, 4, 5, and 6, no statistical tests were performed *a priori* to decide on sample sizes, but sample sizes are consistent with conventions in the field. We used the Mann-Whitney non-parametric test to test for differences between two groups (Figures 1H and 1I, 3D, 3F, and 5D), and the Kruskal-Wallis non-parametric test to test for differences between more than two groups (Figures 1I and 1J). All statistical analysis was performed with GraphPad (Prism). Results of statistical tests are reported in the figure legends.

## Supplementary Material

1

## Figures and Tables

**Table:** KEY RESOURCES TABLE

REAGENT TYPE OR RESOURCE	DESIGNATION	SOURCE OR REFERENCE	IDENTIFIERS	ADDITIONAL INFORMATION
genetic reagent (*D. melanogaster*)	“w[1118]; P{JFRC7-20XUAS-IVS-mCD8::GFP} attp40”	other	N/A	Barret Pfeiffer, Janelia Farm, HHMI
genetic reagent (*D. melanogaster*)	“P{iav-Gal4.K}3”	Bloomington *Drosophila* Stock Center	“RRID:BDSC_52273”	N/A
genetic reagent (*D. melanogaster*)	“10XUASsyn21 Chrimson88-tDT3.1(attP18)”	other	N/A	Allan Wong, Janelia Farm, HHMI
genetic reagent (*D. melanogaster*)	“w[1118],P{13xLexAop-IVS-Syn21-GCaMP6s}”	other	N/A	Allan Wong, Janelia Farm, HHMI
genetic reagent (*D. melanogaster*)	“w[1118] P{y[+t7.7] w[+mC]=hs-FlpG5.PEST}attP3/ w[1118]; +/+; P{y[+t7.7]w[+mC]=10xLexAop(FRT.stop) myr::smGdP-OLLAS} attP2 PBac{y[+mDint2] w[+mC]=10xLexAop(FRT.stop) myr::smGdPHA} VK00005 P{10xLexAop(FRT.stop) myr::smGdP-V5-THS-10xLexAop(FRT.stop) myr::smGdP-FLAG} su(Hw)attP1/+”	other	N/A	Janelia Farm, HHMI
genetic reagent (*D. melanogaster*)	“P{GMR73D10-GAL4} attP2”	Bloomington Drosophila Stock Center	“RRID:BDSC_39819”	N/A
genetic reagent (*D. melanogaster*)	“P{GMR64C04-GAL4} attP2”	Bloomington Drosophila Stock Center	“RRID:BDSC_39296”	N/A
genetic reagent (*D. melanogaster*)	“P{y[+t7.7] w[+mC] =20xUAS-IVSjGCaMP7f}VK00005”	Bloomington Drosophila Stock Center	“RRID:BDSC_79031”	N/A
genetic reagent (*D. melanogaster*)	“w[1118]; P{y[+t7.7] w[+mC]=13xLexAop2-IVS-GCaMP6f-p10} su(Hw)attP5”	Bloomington Drosophila Stock Center	“RRID:BDSC_44277”	N/A
genetic reagent (*D. melanogaster*)	“*trans*-Tango”	Bloomington Drosophila Stock Center	“RRID:BDSC_77479	N/A
genetic reagent (*D. melanogaster*)	“w[1118];P{VT000629-LexA} attp40”	other	N/A	Barry Dickson, Janelia Farm, HHMI
genetic reagent (*D. melanogaster*)	“w[1118];P{VT008498-LexA} attp40”	other	N/A	Barry Dickson, Janelia Farm, HHMI
genetic reagent (*D. melanogaster*)	“w[1118];P{VT059469-LexA} attp40”	other	N/A	Barry Dickson, Janelia Farm, HHMI
genetic reagent (*D. melanogaster*)	“w[1118];P{VT000629-LexA} attp40”	other	N/A	Barry Dickson, Janelia Farm, HHMI
genetic reagent (*D. melanogaster*)	“w[1118];P{GMR79C08-LexA} attp40”	other	“RRID:BDSC_54369”	Gerald M. Rubin Janelia Farm, HHMI
genetic reagent (*D. melanogaster*)	“w[1118];P{GMR79E01-LexA} attp40”	other	“RRID:BDSC_54677”	Gerald M. Rubin Janelia Farm, HHMI
genetic reagent (*D. melanogaster*)	“w[1118];P{GMR74B06-LexA} attp40”	other	“RRID:BDSC_54116”	Gerald M. Rubin Janelia Farm, HHMI
genetic reagent (*D. melanogaster*)	“w[1118];P{GMR34A09-LexA} attp40”	other	“RRID:BDSC_54290”	Gerald M. Rubin Janelia Farm, HHMI
genetic reagent (*D. melanogaster*)	“w[1118];P{GMR09B05-LexA} attp40”	other	N/A	Gerald M. Rubin Janelia Farm, HHMI
genetic reagent (*D. melanogaster*)	“w[1118];P{VT037652-LexA} attp40”	other	N/A	Barry Dickson, Janelia Farm, HHMI
genetic reagent (*D. melanogaster*)	“w[1118];P{VT008170-LexA} attp40”	other	N/A	Barry Dickson, Janelia Farm, HHMI
genetic reagent (*D. melanogaster*)	“w[1118];P{GMR65C07-LexA} attp40”	other	N/A	Gerald M. Rubin Janelia Farm, HHMI
genetic reagent (D. melanogaster)	“w[1118];P{GMR18H03-LexA} attp40”	other	N/A	Gerald M. Rubin Janelia Farm, HHMI
genetic reagent (*D. melanogaster*)	“w[1118];P{GMR64F10-LexA} attp40”	other	“RRID:BDSC_54912”	Gerald M. Rubin Janelia Farm, HHMI
genetic reagent (*D. melanogaster*)	“w[1118];P{GMR13E04-LexA} attp40”	other	“RRID:BDSC_52457”	Gerald M. Rubin Janelia Farm, HHMI
genetic reagent (D. melanogaster)	“w[1118];P{VT043132-LexA} attp40”	other	N/A	Barry Dickson, Janelia Farm, HHMI
genetic reagent (D. melanogaster)	“w[1118];P{GMR26H12-LexA} attp40”	other	“RRID:BDSC_54405”	Gerald M. Rubin Janelia Farm, HHMI
genetic reagent (D. melanogaster)	“w[1118];P{VT006903-LexA} attp40”	other	N/A	Barry Dickson, Janelia Farm, HHMI
genetic reagent (*D. melanogaster*)	“w[1118];P{VT034765-LexA} attp40”	other	N/A	Barry Dickson, Janelia Farm, HHMI
genetic reagent (*D. melanogaster*)	“w[1118];P{VT029362-LexA} attp40”	other	N/A	Barry Dickson, Janelia Farm, HHMI
genetic reagent (*D. melanogaster*)	“w[1118];P{GMR46H07-LexA} attp40”	other	“RRID:BDSC_61549”	Gerald M. Rubin Janelia Farm, HHMI
genetic reagent (D. melanogaster)	“w[1118];P{GMR14B11-LexA} attp40”	other	“RRID:BDSC_52469”	Gerald M. Rubin Janelia Farm, HHMI
genetic reagent (D. melanogaster)	“w[1118];P{VT044964-LexA} attp40”	other	N/A	Barry Dickson, Janelia Farm, HHMI
genetic reagent (D. melanogaster)	“w[1118];P{VT006555-LexA} attp40”	other	N/A	Barry Dickson, Janelia Farm, HHMI
genetic reagent (D. melanogaster)	“w[1118];P{GMR10E06-LexA} attp40”	other	“RRID:BDSC_52417”	Gerald M. Rubin Janelia Farm, HHMI
genetic reagent (D. melanogaster)	“w[1118];P{GMR24G06-LexA} attp40”	other	“RRID:BDSC_53550”	Gerald M. Rubin Janelia Farm, HHMI
genetic reagent (*D. melanogaster*)	“w[1118];P{GMR37G12-LexA} attp40”	other	“RRID:BDSC_52765”	Gerald M. Rubin Janelia Farm, HHMI
genetic reagent (*D. melanogaster*)	“w[1118];P{GMR13D05-LexA} attp40”	other	“RRID:BDSC_52456”	Gerald M. Rubin Janelia Farm, HHMI
genetic reagent (*D. melanogaster*)	“w[1118];P{GMR53B02-P65.AD} attp40/+; P{GMR64D09-Gal4.DBD} attp2/+”	this study	N/A	Gerald M. Rubin Janelia Farm, HHMI
genetic reagent (*D. melanogaster*)	“w[1118];P{VT020600-P65.AD} attp40/+; P{GMR75G05-Gal4.DBD} attp2/+”	this study	N/A	Gerald M. Rubin, Barry Dickson, Janelia Farm, HHMI
genetic reagent (*D. melanogaster*)	“w[1118];P{VT018774-P65.AD} attp40/+; P{GMR21D12-Gal4} attp2/+”	this study	N/A	Gerald M. Rubin, Barry Dickson, Janelia Farm, HHMI
genetic reagent (*D. melanogaster*)	“w[1118]; P{VT018774-P65.AD} attp40/+; P {VT040547-Gal4.DBD} attp2/+”	this study	N/A	Barry Dickson, Janelia Farm, HHMI
genetic reagent (*D. melanogaster*)	“w[1118];P{VT020600-P65.AD} attp40/+; P{GMR75G05-Gal4.DBD} attp2/+”	this study	N/A	Gerald M. Rubin, Barry Dickson, Janelia Farm, HHMI
genetic reagent (*D. melanogaster*)	“w[1118];P{GMR92D04-P65.AD} attp40/+; P{GMR59A06-Gal4.DBD} attp2/+”	this study	N/A	Gerald M. Rubin Janelia Farm, HHMI
antibody	nc82 (mouse monoclonal)	Developmental Studies Hybridoma Bank	RRID: AB_2314866	N/A
antibody	Rabbit polyclonal α-GFP	Life Technologies	RRID: AB_221569	N/A
antibody	AF568 Goat α-Mouse	Life Technologies	RRID: AB_143157	N/A
	AF488 Goat α-Rabbit	Life Technologies	RRID: AB_2536097	N/A
antibody	rabbit polyclonal anti-HA	Cell Signaling Technologies	RRID: AB_1549585	N/A
antibody	rat monoclonal anti-FLAG	Novus Bio	RRID: AB_1625982	N/A
antibody	mouse polyclonal anti-V5:DyLight 550	AbD Serotec	RRID: AB_2687576	N/A
antibody	Cy2 Goat α-Mouse	Jackson Immuno Research	RRID: AB_2338746	N/A
antibody	AF594 Donkey α-Rabbit	Jackson Immuno Research	RRID: AB_2340621	N/A
antibody	ATTO 647N Goat α-Rat IgG (H&L) Antibody	Rockland	605-456-013S	“”
chemical compound	methyllycaconitine (MLA)	Tocris	TOCRIS_1029	“1μM”
chemical compound	picrotoxin (PTX)	Sigma-Aldrich	P1675	“10μM”
chemical compound	all trans-retinal powder	Sigma-Aldrich	R2500	“0.2μM”
software, algorithm	MATLAB	Mathworks	“RRID:SCR_001622”	N/A
software, algorithm	FIJI	“PMID:22743772”	“RRID:SCR_002285”	N/A
software, algorithm	ScanImage 5.2	Vidrio Technologies	“RRID:SCR_014307”	N/A
software, algorithm	VVDviewer	N/A	N/A	https://github.com/takashi310/VVD_Viewer

## Highlights

- A map of functional connectivity between leg proprioceptors and downstream neurons
- Joint position and directional movement signals converge in second-order neurons
- Second-order neurons integrate tibia vibration signals across legs
- Inhibition gates and sculpts neural activity in central proprioceptive pathways
