# Supplementary material for: Functional architecture of neural circuits for leg proprioception in Drosophila
Source: Curr Biol. Author manuscript; Available in PMC 2021 Dec 11. (PMC8665017; doi:10.1016/j.cub.2021.09.035)
Supplement: 1 [file NIHMS1748727-supplement-1.pdf]

Current Biology, Volume *31*

Supplemental Information

Functional architecture of neural circuits

for leg proprioception in *Drosophila*

Chenghao Chen, Sweta Agrawal, Brandon Mark, Akira Mamiya, Anne Sustar, Jasper S. Phelps, Wei-Chung Allen Lee, Barry J. Dickson, Gwyneth M. Card, and John C. Tuthill

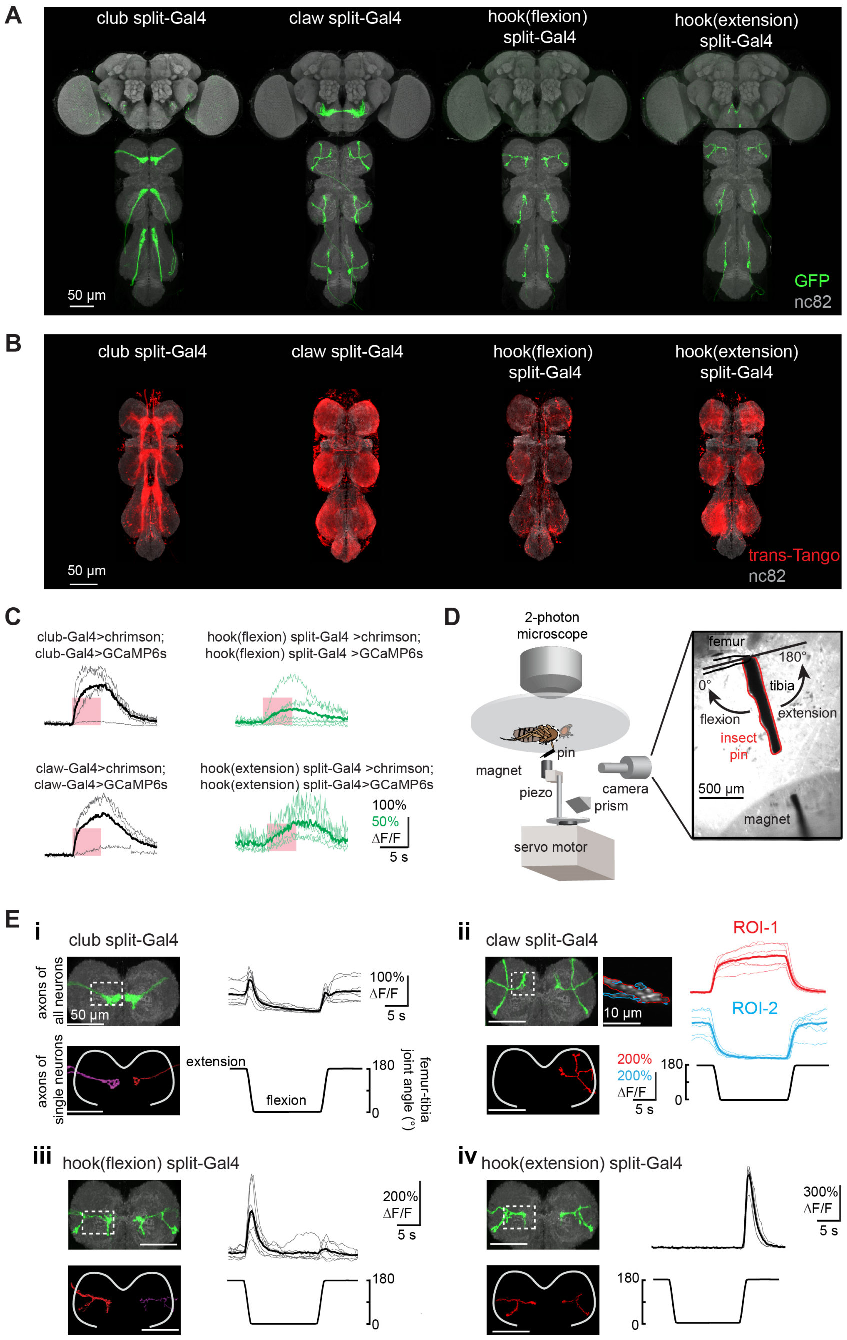

Figure S1. Split-Gal4 lines for targeting subtypes of femoral chordotonal organ (FeCO) proprioceptors. Related to Figure 1. (**A**) GFP (green) expression in VNC and brain driven by split-Gal4 lines targeting subtypes of FeCO neurons. Grey: neuropil of VNCs and brains were stained with nc82. **(B)** Post-synaptic partners of four subclasses FeCO neurons revealed by trans-Tango. **(C)** Optogenetic stimulation of FeCO axons increases their calcium activity. Changes of GCaMP6s fluorescence relative to baseline (ΔF/F) in the axons of each FeCO subtypes to their self-stimulation. The thick lines in each panel represent average values. The pink windows indicate stimulus duration (5 seconds, laser power= 0.28 mW/mm^2). (**D**) Experimental set-up for recording proprioceptive tuning of FeCO axons. (**E**) Anatomy and proprioceptive tuning of FeCO neurons labeled by four split-Gal4 lines. (i) Left: GFP labelled populations (upper panel) and single axon (lower panel) of club axons labeled by a split-Gal4 line. Grey: neuropil stained with nc82. Right: club neurons respond to bidirectional movement phasically. Tonic response at 180° caused by active tibia vibration at tibia fully extension. Changes of GCaMP7f fluorescence relative to baseline (ΔF/F) recorded from the regions outlined in a white rectangle at left when swung the tibia at 360°/s (n=8). (ii) Same as i, but for claw neurons responding tonically to tibia movement (n=7). Two sub branches could be further separated in response to flexion (ROI-1) and extension (ROI2) (iii) Same as (i), but for hook (flexion) neurons that respond phasically to tibia flexion (n=7). (iv) Same as (i), but for hook (extension) neurons that phasically respond to tibia extension (n=6).

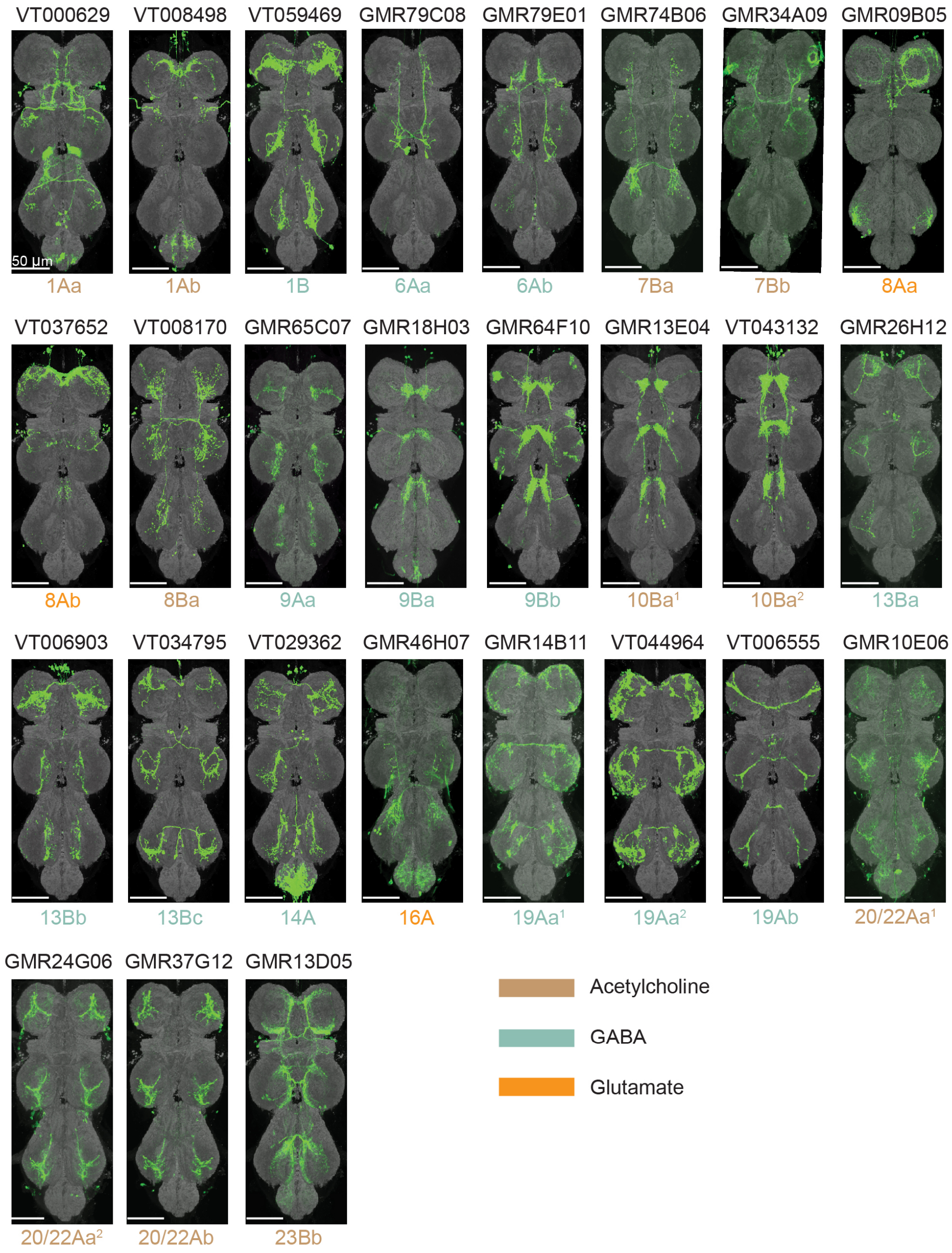

Figure S2. A collection of LexA driver lines used for functional connectivity experiments in this study. Related to Figure 1.

GFP (green) was expressed in the VNC driven by indicated LexA lines. Anatomy was used to determine the lineage identity described below each VNC image. The colors for each lineage and FeCO subtype indicate the putative neurotransmitter that they release.

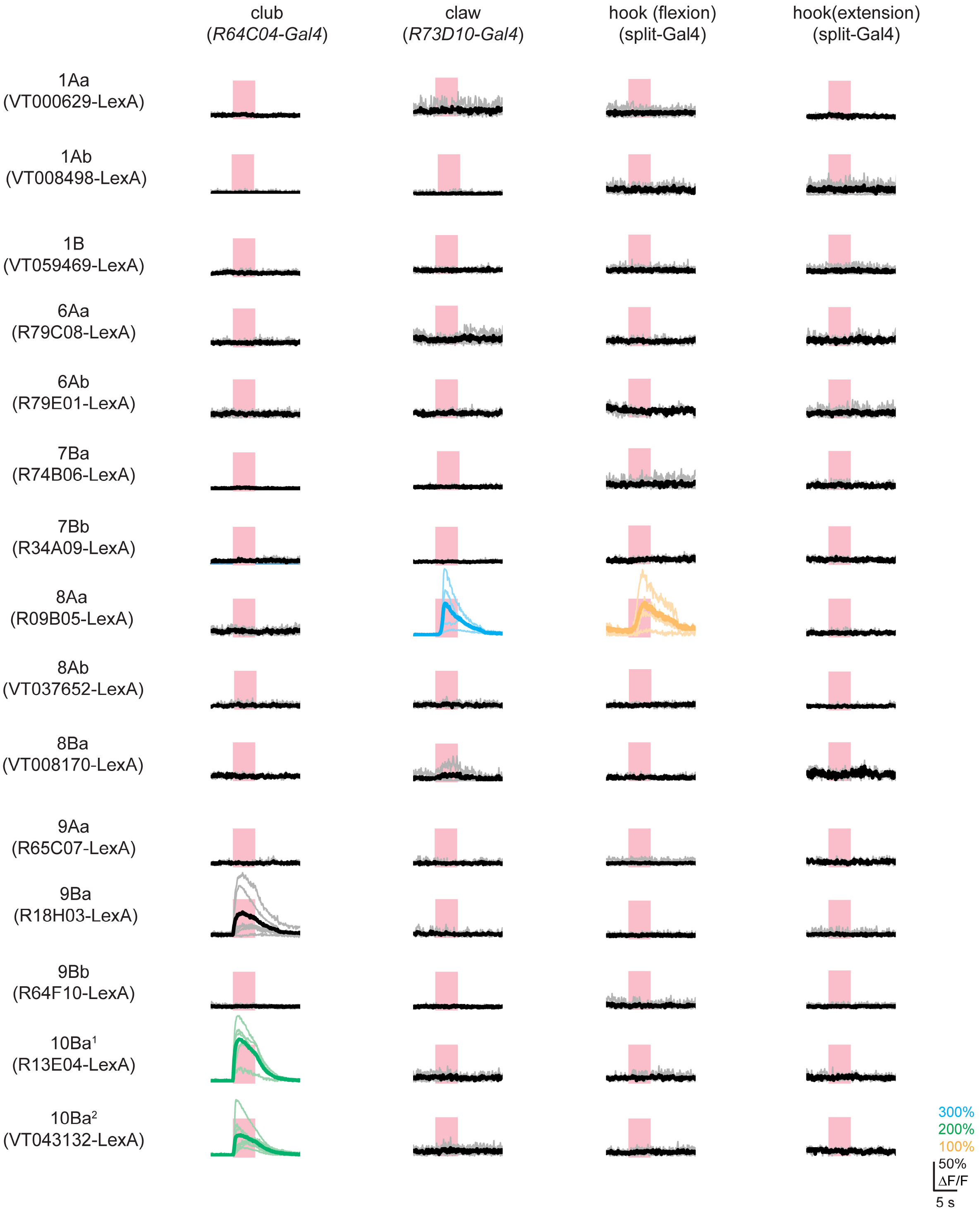

Figure S3. Times series data from functional connectivity experiments. Related to Figure 1. Changes of GCaMP6s fluorescence relative to baseline (ΔF/F) were recorded in each driver line in response to optogenetic stimulation of four FeCO subtypes. The pink windows indicate stimulus duration (5 seconds, laser power= 0.28 mW/mm^2).

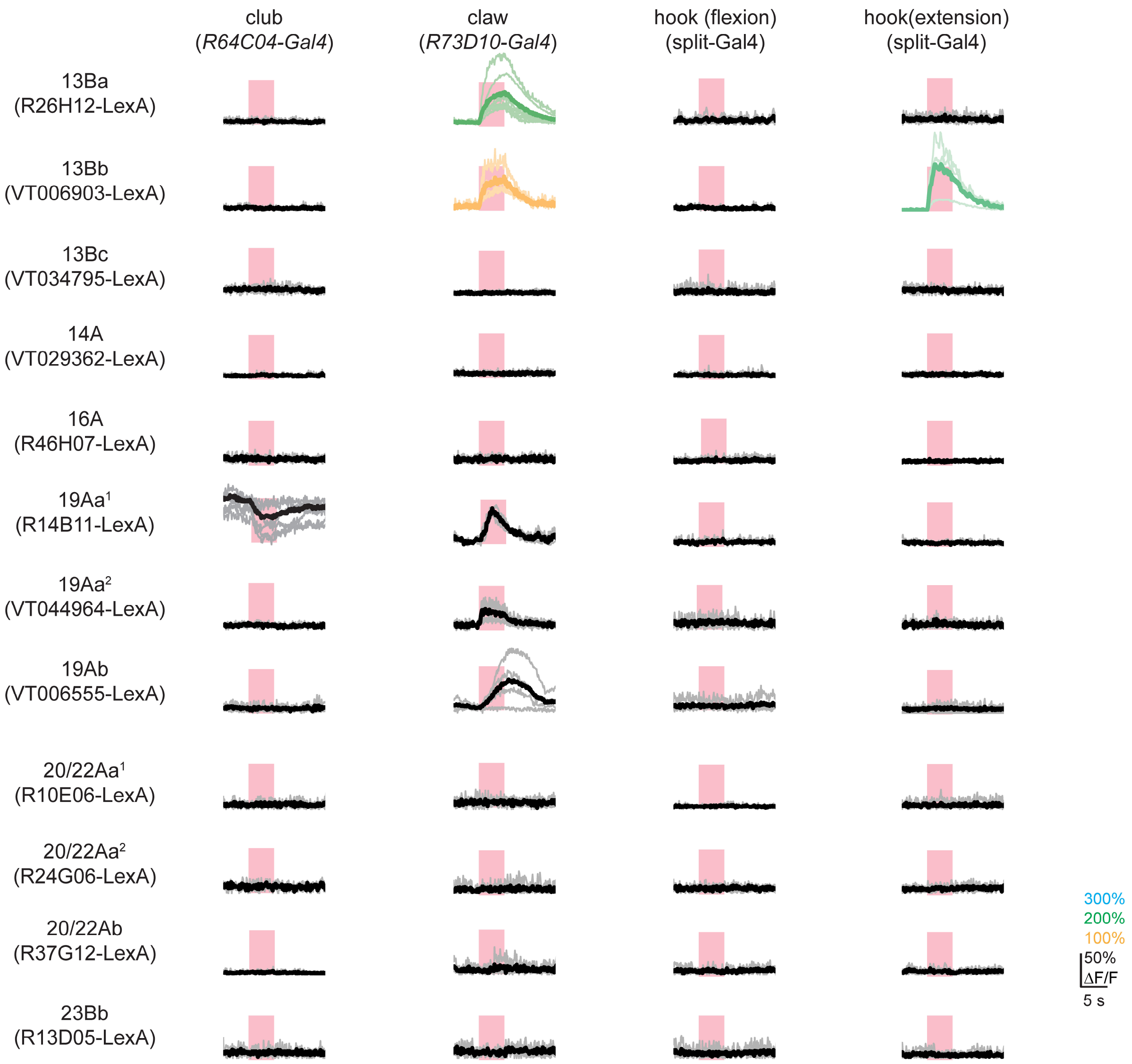

Figure S4. Times series data from functional connectivity experiments (continued from Figure S3.). Related to Figure 1.

Changes of GCaMP6s fluorescence relative to baseline (ΔF/F) were recorded in each driver line in response to optogenetic stimulation of four FeCO subtypes. The pink windows indicate stimulus duration (5 seconds, laser power= 0.28 mW/mm^2).

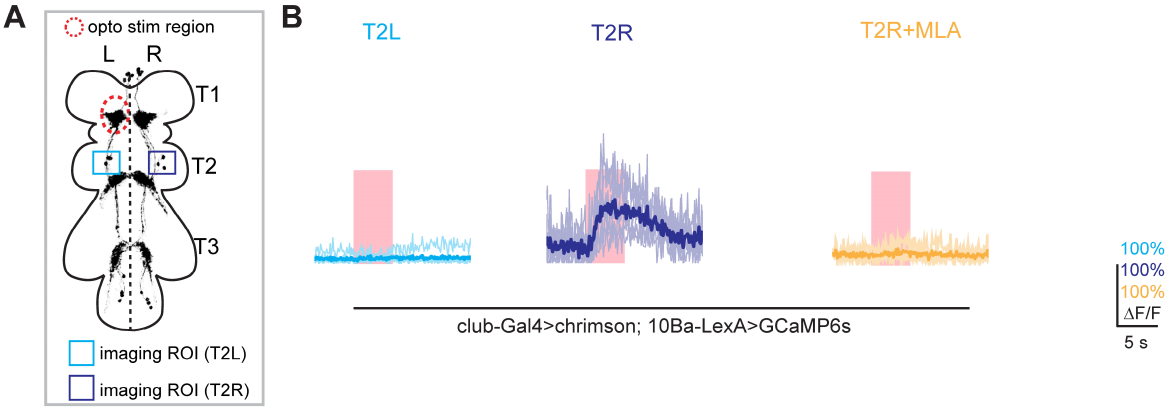

Figure S5. 10Ba neurons in T2R integrate proprioceptive stimuli from anterior, contralateral club neurons via 10Ba neurons. Related to Figure 3.

(**A**) Schematic of focal stimulation while imaging calcium signals of the 10Ba soma in T2. (**B**) Calcium responses were observed in 10Ba in left (T2L) but not in right (T2R) neuromeres in respond to club neurons from T1L, and signals could be blocked by applying MLA (T2R+MLA). The pink windows indicate stimulus duration (5 seconds, laser power= 0.28 mW/mm^2).

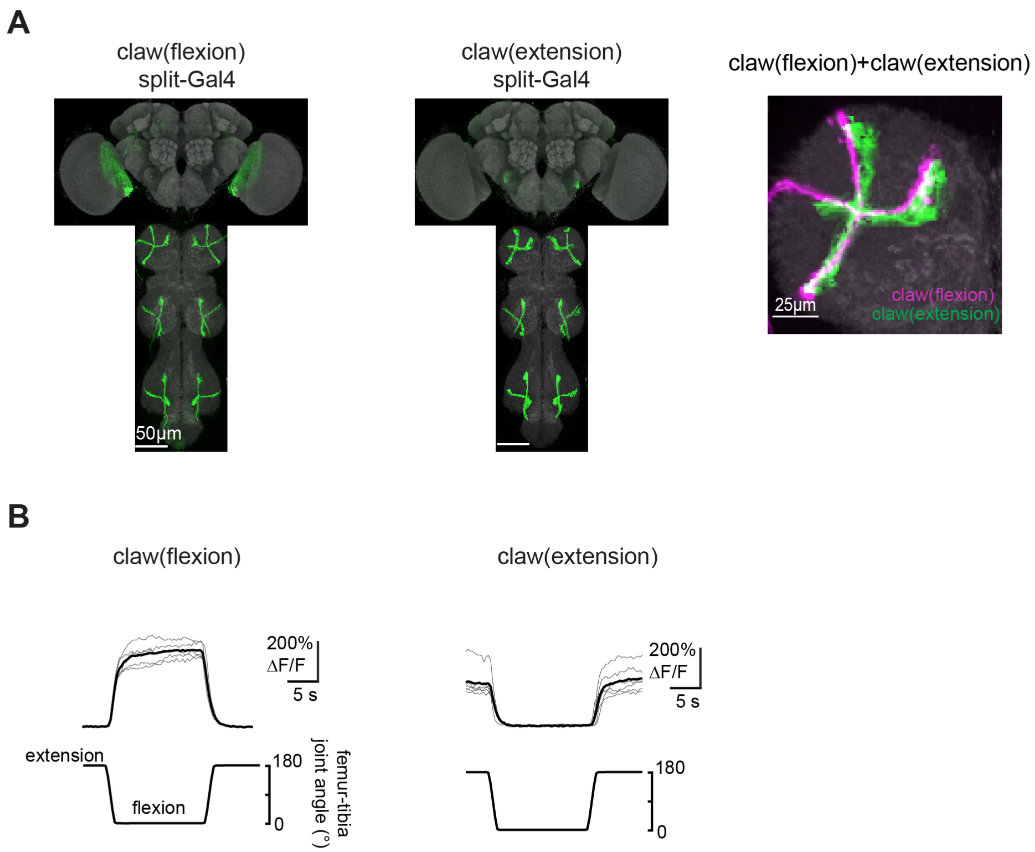

Figure S6. Distinct classes of claw neurons respond to tibia flexion and extension. Related to Figure 4. (**A**) Genetic driver lines labeling claw neurons that encode tibia flexion and extension. GFP (green) expression in VNC and brain driven by split-Gal4 lines targeting subtypes of the claw neurons. Grey: neuropils and brains were stained with nc82. Right: co-localization of claw(flexion) and claw(extension) neurons. VNC images were aligned to a common template *in silico*. (**B**) Calcium responses of claw(flexion) and claw(extension) neurons during passive movement of the tibia (n=6 flies of each genotype).

	club	claw	hook (flexion)	hook (extension)
cell numbers labeled by split-Gal4 lines	30	28	5	6
cell numbers identified by Functional connectivity (FC)	147	443	21	89
cell numbers identified by trans-Tango (TT)	216	566	74	197
coverage ratio (N_{FC}/N_{TT})	0.68	0.78	0.28	0.45

Table S1. Comparison of cell numbers that identified by functional connectivity and trans-Tango. Related to Figure 1 and STAR Methods.

post- / pre-	9Ba_1_T1L*	9Ba_1_T1R*	10Ba_2_T2R	10Ba_1_T1R	10Ba_3_T1L	13Bb_1_T1R*
**claw_1_T1L***	**0**	**0**	**0**	**0**	**0**	**0**
**claw_2_T1L***	**0**	**0**	**0**	**0**	**0**	**1**
**hook (extension)_1**	**0**	**0**	**0**	**0**	**0**	**29**
**club_1_T1L***	**7**	**2**	**0**	**0**	**0**	**0**
**club_2_T1L**	**0**	**2**	**0**	**0**	**0**	**0**
**club_3_T1L**	**0**	**0**	**0**	**3**	**0**	**0**
**club_4_T1L**	**0**	**0**	**0**	**0**	**0**	**0**
**club_5_T1L***	**4**	**1**	**0**	**0**	**0**	**0**
**club_6_T1L**	**0**	**2**	**0**	**0**	**7**	**0**
**club_7_T1L**	**0**	**1**	**0**	**0**	**0**	**0**
**club_8_T1L**	**0**	**1**	**0**	**0**	**0**	**0**
**10Ba_3_T1L**	**0**	**0**	**3**	**0**	**0**	**0**

Table S2. Numbers of synapses identified between sensory (pre) and second-order (post) proprioceptive neurons by EM reconstruction. Related to Figures 2-4 and STAR Methods.

* fully traced cells

Figure 1A	w[1118]; P{JFRC7-20XUAS-IVS-mCD8::GFP} attp40/+; iav-Gal4/+
Figure1B	w[1118], P{13xLexAop-IVS-Syn21-GCaMP6s}, P{20xUAS-IVS-Chrimson::tdTomato}/+; P{GMR13E04-LexA} attp40/+;<br>P{GMR64C04-Gal4} attp2/+
Figure 1C*	w[1118], P{13xLexAop-IVS-Syn21-GCaMP6s},P{20xUAS-IVS-Chrimson::tdTomato}/+ ; P{VT000629-LexA} attp40/+;<br>P{GMR64C04-Gal4} attp2/+<br>w[1118], P{13xLexAop-IVS-Syn21-GCaMP6s}, P{20xUAS-IVS-Chrimson::tdTomato}/+ ; P{VT000629-LexA} attp40/+;<br>P{GMR73D10-Gal4} attp2/+<br>w[1118], P{13xLexAop-IVS-Syn21-GCaMP6s}, P{20xUAS-IVS-Chrimson::tdTomato}/+; P{VT000629-LexA}<br>attp40/P{VT018774-P65.AD} attp40; P{GMR32H08-Gal4.DBD} attp2/+<br>w[1118], P{13xLexAop-IVS-Syn21-GCaMP6s}, P{20xUAS-IVS-Chrimson::tdTomato}/+; P{VT000629-LexA }<br>attp40/P{VT018774-P65.AD} attp40; P{VT040547-Gal4.DBD} attp2/+
Figure 1D	w[1118], P{13xLexAop-IVS-Syn21-GCaMP6s}, P{20xUAS-IVS-Chrimson::tdTomato}/+ ; P{GMR13E04-LexA} attp40/+;<br>P{GMR64C04-Gal4} attp2/+<br>w[1118], P{13xLexAop-IVS-Syn21-GCaMP6s}, P{20xUAS-IVS-Chrimson::tdTomato}/+ ; P{GMR26H12-LexA} attp40/+;<br>P{GMR73D10-Gal4} attp2/+<br>w[1118], P{13xLexAop-IVS-Syn21-GCaMP6s}, P{20xUAS-IVS-Chrimson::tdTomato}/+; P{GMR09B05-LexA}<br>attp40/P{VT018774-P65.AD} attp40; P{GMR32H08-Gal4.DBD} attp2/+<br>w[1118], P{13xLexAop-IVS-Syn21-GCaMP6s}, P{20xUAS-IVS-Chrimson::tdTomato}/+ ; P{VT006903-LexA}<br>attp40/P{VT018774-P65.AD} attp40; P{VT040547-Gal4.DBD} attp2/+
Figure2A	w[1118]; P{JFRC7-13xLexAop-IVS-mCD8::GFP} attp40/P{GMR18H03-LexA} attp40; +/+<br>PBac{hsFlp2::PEST}attP3/+; P{GMR18H03-LexA} attp40/+; P{JFRC201-10xLexAop-FRT>STOP>FRT-myr::smGFP-<br>HA}VK00005, P{JFRC240-10xLexAop-FRT>STOP>FRT-myr::smGFP-V5}, P{10xLexAop-FRT>STOP>FRT-myr::smGFP-<br>FLAG}su(Hw)attP1/+
Figure 2C	w[1118], P{13xLexAop-IVS-Syn21-GCaMP6s},P{20xUAS-IVS-Chrimson::tdTomato}/+; P{GMR18H03-LexA}<br>attp40/P{GMR53B02-P65.AD} attp40; P{GMR64D09-Gal4.DBD}attp2/+
Figure 2G-H	w[1118]; P{13xLexAop2-IVS-GCaMP6s}attp5/P{GMR18H03-LexA}attp40/+;<br>PBac{y[+mDint2] w[+mC]=13XLexAop2-IVS-tdTomato.nls}VK00022/+
Figure 3A	w[1118]; P{JFRC7-13xLexAop-IVS-mCD8::GFP}attp40/P{GMR13E04-LexA} attp40; +/+<br>PBac{hsFlp2::PEST}attP3/+; P{GMR13E04-LexA}attp40/+; P{JFRC201-10xLexAop-FRT>STOP>FRT-myr::smGFP-<br>HA}VK00005, P{JFRC240-10xLexAop-FRT>STOP>FRT-myr::smGFP-V5}, P{10xLexAop-FRT>STOP>FRT-myr::smGFP-<br>FLAG}su(Hw)attP1/+
Figure 3 C-F	w[1118], P{13xLexAop-IVS-Syn21-GCaMP6s}, P{20xUAS-IVS-Chrimson::tdTomato}/+; P{GMR13E04-LexA}<br>attp40/P{GMR53B02-P65.AD}attp40; P{GMR64D09-Gal4.DBD} attp2/+
Figure 3 G	w[1118], P{13xLexAop-IVS-Syn21-GCaMP6s},P{20xUAS-IVS-Chrimson::tdTomato}/+; P{GMR13E04-LexA}<br>attp40/P{GMR53B02-P65.AD} attp40; P{GMR64D09-Gal4.DBD}attp2/+<br>w[1118], P{20xUAS-IVS-Chrimson::tdTomato},P{20xUAS-IVS-syn21-GCaMP6s}/+ ; P{GMR53B02-P65.AD} attp40/+;<br>P{GMR64D09-Gal4.DBD} attp2/+
Figure 3 J-K	w[1118]; P{GMR13E04-LexA} attp40/+;P{13XLexAop2-IVS-GCaMP6f-p10} su(Hw) attP5/PBac{y[+mDint2]<br>w[+mC]=13XLexAop2-IVS- tdTomato.nls}VK00022/+
Figure 4A	w[1118]; P{JFRC7-13xLexAop-IVS-mCD8::GFP} attp40/P{VT006903-LexA} attp40/+; +/+<br>PBac{hsFlp2::PEST}attP3/+; P{VT006903-LexA}attp40/+; P{JFRC201-10xLexAop-FRT>STOP>FRT-myr::smGFP-<br>HA}VK00005, P{JFRC240-10xLexAop-FRT>STOP>FRT-myr::smGFP-V5}, P{10xLexAop-FRT>STOP>FRT-myr::smGFP-<br>FLAG}su(Hw)attP1/+
Figure 4C	w[1118],P{13xLexAop-IVS-Syn21-GCaMP6s},P{20xUAS-IVS-Chrimson::tdTomato; P{VT006903-LexA} attp40/<br>P{VT018774-P65.AD} attp40; P{VT040547-Gal4.DBD} attp2/+<br>w[1118],P{13xLexAop-IVS-Syn21-GCaMP6s},P{20xUAS-IVS-Chrimson::tdTomato/+; P{VT006903-LexA}<br>attp40/P{VT020600-P65.AD} attp40; P{GMR75G05-Gal4.DBD} attp2/+
Figure 4D	w[1118],P{13xLexAop-IVS-Syn21-GCaMP6s},P{20xUAS-IVS-Chrimson::tdTomato/+; P{VT006903-LexA}<br>attp40/P{GMR92D04-P65.AD} attp40; P{GMR59A06-Gal4.DBD} attp2/+<br>w[1118],P{13xLexAop-IVS-Syn21-GCaMP6s},P{20xUAS-IVS-Chrimson::tdTomato/+; P{VT006903-LexA}<br>attp40/P{VT017745-P65.AD} attp40; P{GMR55C05-Gal4.DBD} attp2/+
Figure 4E	w[1118]; P{VT006903-LexA} attp40/+; P{13XLexAop2-IVS-GCaMP6f-p10} su(Hw)attP5/ PBac{y[+mDint2]<br>w[+mC]=13XLexAop2-IVS-tdTomato.nls}VK00022/+
Figure 5A	w [1118], P{13xLexAop-IVS-Syn21-GCaMP6s},P{20xUAS-IVS-Chrimson::tdTomato/+; P{GMR64F10-<br>LexA}attp40/P{GMR53B02-P65.AD}attp40; P{GMR64D09-Gal4.DBD} attp2/+<br>w [1118], P{13xLexAop-IVS-Syn21-GCaMP6s},P{20xUAS-IVS-Chrimson::tdTomato/+; P{GMR37G12-<br>LexA}attp40/P{VT020600-P65.AD} attp40; P{GMR75G05-Gal4.DBD} attp2/+
Figure 5B-D	w [1118], P{13xLexAop-IVS-Syn21-GCaMP6s},P{20xUAS-IVS-Chrimson::tdTomato/+; P{GMR13E04-LexA}<br>attp40/P{GMR53B02-P65.AD} attp40; P{ GMR64D09-Gal4.DBD} attp2/+

	w [1118], P{13xLexAop-IVS-Syn21-GCaMP6s},P{20xUAS-IVS-Chrimson::tdTomato/+; P{GMR26H12-LexA} attp40/P{VT020600-P65.AD} attp40; P{ GMR75G05-Gal4.DBD} attp2/+<br>w [1118], P{13xLexAop-IVS-Syn21-GCaMP6s},P{20xUAS-IVS-Chrimson::tdTomato/+; P{VT006903-LexA} attp40/P{VT018774-P65.AD} attp40; P{VT040547-Gal4.DBD} attp2/+<br>w [1118], P{20xUAS-IVS-Syn21-GCaMP6s},P{20xUAS-IVS-Chrimson::tdTomato}/+; P{GMR37G12-LexA} attp40/P{VT020600-P65.AD} attp40; P{ GMR75G05-Gal4.DBD} attp2/+
Figure S1A	w[1118]; P{JFRC7-20xUAS-IVS-mCD8::GFP} attp40/+; P{GMR53B02-P65.AD} attp40/+; P{GMR64D09-Gal4.DBD} attp2/+<br>w[1118]; P{JFRC7-20xUAS-IVS-mCD8::GFP} attp40/+; P{VT020600-P65.AD} attp40/+; P{ GMR75G05-Gal4.DBD} attp2/+<br>w[1118]; P{JFRC7-20xUAS-IVS-mCD8::GFP} attp40/+; P{GMR70H02 -P65.AD} attp40/+; P{ GMR32H08-Gal4.DBD}attp2/+<br>w[1118]; P{JFRC7-20xUAS-IVS-mCD8::GFP} attp40/+; P{VT018774-P65.AD} attp40/+; P{VT040547-Gal4.DBD} attp2/+
Figure S1B	w [1118], P{UAS-myr-GFP}, P{QUAS-mtdTomato(3xHA)/+; P{GMR53B02-P65.AD} attp40/*trans*-Tango; P{GMR64D09-Gal4.DBD} attp2/+<br>w [1118], P{UAS-myr-GFP}, P{QUAS-mtdTomato(3xHA)/+; P{VT020600-P65.AD} attp40/*trans*-Tango; P{ GMR75G05-Gal4.DBD} attp2/+<br>w [1118], P{UAS-myr-GFP}, P{QUAS-mtdTomato(3xHA)/+; P{GMR70H02 -P65.AD} attp40/*trans*-Tango; P{ GMR32H08-Gal4.DBD} attp2/+<br>w [1118], P{UAS-myr-GFP}, P{QUAS-mtdTomato(3xHA)/+; P{VT018774-P65.AD} attp40/*trans*-Tango; P{VT040547-Gal4.DBD} attp2/+
Figure S1C	w [1118], P{20xUAS-IVS-Syn21-GCaMP6s}, P{20xUAS-IVS-Chrimson::tdTomato/+; P{GMR53B02-P65.AD} attp40/+; P{GMR64D09-Gal4.DBD} attp2/+<br>w [1118], P{20xUAS-IVS-Syn21-GCaMP6s}, P{20xUAS-IVS-Chrimson::tdTomato}/+; P{VT020600-P65.AD} attp40/+; P{ GMR75G05-Gal4.DBD} attp2/+<br>w [1118], P{20xUAS-IVS-Syn21-GCaMP6s}, P{20xUAS-IVS-Chrimson::tdTomato}/+; P{GMR70H02 -P65.AD} attp40/+; P{ GMR32H08-Gal4.DBD} attp2/+<br>w [1118], P{20xUAS-IVS-Syn21-GCaMP6s}, P{20xUAS-IVS-Chrimson::tdTomato}/+; P{VT018774-P65.AD} attp40/+; P{VT040547-Gal4.DBD} attp2/+
Figure S1E	w [1118]; P{GMR53B02-P65.AD} attp40/ P{UAS-tdTomato}2; P{GMR64D09-Gal4.DBD} attp2/PBac{y[+t7.7] w[+mC]=20XUAS-IVS-jGCaMP7f}VK00005<br>w [1118]; P{VT020600-P65.AD} attp40/P{UAS-tdTomato}2; P{ GMR75G05-Gal4.DBD} attp2/PBac{y[+t7.7] w[+mC]=20XUAS-IVS-jGCaMP7f}VK00005<br>w [1118]; P{GMR70H02 -P65.AD} attp40/P{UAS-tdTomato}2; P{ GMR32H08-Gal4.DBD} attp2/PBac{y[+t7.7] w[+mC]=20XUAS-IVS-jGCaMP7f}VK00005<br>w [1118]; P{VT018774-P65.AD} attp40/P{UAS-tdTomato}2; P{VT040547-Gal4.DBD} attp2/PBac{y[+t7.7] w[+mC]=20XUAS-IVS-jGCaMP7f}VK00005
Figure S3-4*	w[1118], P{13xLexAop-IVS-Syn21-GCaMP6s},{20xUAS-IVS-Chrimson::tdTomato}; P{VT000629-LexA} attp40/+; P{GMR64C04-Gal4} attp2/+<br>w[1118], P{13xLexAop-IVS-Syn21-GCaMP6s},P{20xUAS-IVS-Chrimson::tdTomato}; P{VT000629-LexA} attp40/+; P{GMR73D10-Gal4} attp2/+<br>w[1118], P{13xLexAop-IVS-Syn21-GCaMP6s},P{20xUAS-IVS-Chrimson::tdTomato}; P{VT000629-LexA} attp40/P{VT018774-P65.AD}attp40; P{GMR32H08-Gal4.DBD} attp2/+<br>w[1118], P{13xLexAop-IVS-Syn21-GCaMP6s},P{20xUAS-IVS-Chrimson::tdTomato}; P{VT000629-LexA }attp40/P{VT018774-P65.AD} attp40; P{VT040547-Gal4.DBD} attp2/+
Figure S5B	w[1118], P{13xLexAop-IVS-Syn21-GCaMP6s}, P{20xUAS-IVS-Chrimson::tdTomato}/+; P{GMR13E04-LexA} attp40/P{GMR53B02-P65.AD}attp40; P{GMR64D09-Gal4.DBD} attp2/+
Figure S6A	w[1118]; P{JFRC7-20xUAS-IVS-mCD8::GFP} attp40/ P{GMR92D04-P65.AD} attp40; P{GMR59A06-Gal4.DBD}attp2/+<br>w[1118]; P{JFRC7-20xUAS-IVS-mCD8::GFP} attp40/P{VT020600-P65.AD} attp40; P{GMR75G05-Gal4.DBD}attp2/+
Figure S6B	w [1118]; P{GMR92D04-P65.AD} attp40/ P{UAS-tdTomato}2; P{GMR59A06-Gal4.DBD} attp2/PBac{y[+t7.7] w[+mC]=20XUAS-IVS-jGCaMP7f}VK00005<br>w [1118]; P{ VT020600-P65.AD} attp40/ P{UAS-tdTomato}2; P{ GMR75G05-Gal4.DBD} attp2/PBac{y[+t7.7] w[+mC]=20XUAS-IVS-jGCaMP7f}VK00005
*: the underlined genotypes are different for LexA lines labelling different VNC neurons. The LexA lines for each lineage studied in this paper are listed in Figure S2.	

Table S3. Table of Genotypes. Related to STAR Methods.
